# Potential of Non-Contact Dynamic Response Measurements for Predicting Small Size or Hidden Damages in Highly Damped Structures

**DOI:** 10.3390/s24185871

**Published:** 2024-09-10

**Authors:** Zakrya Azouz, Barmak Honarvar Shakibaei Asli, Muhammad Khan

**Affiliations:** Centre for Life-Cycle Engineering and Management, Faculty of Engineering and Applied Sciences, Cranfield University, Cranfield, Bedfordshire MK43 0AL, UK

**Keywords:** dynamic response, 3D printing, fused deposition modelling, crack depth, image processing, damping ratio, signal processing

## Abstract

Vibration-based structural health monitoring (SHM) is essential for evaluating structural integrity. Traditional methods using contact vibration sensors like accelerometers have limitations in accessibility, coverage, and impact on structural dynamics. Recent digital advancements offer new solutions through high-speed camera-based measurements. This study explores how camera settings (speed and resolution) influence the accuracy of dynamic response measurements for detecting small cracks in damped cantilever beams. Different beam thicknesses affect damping, altering dynamic response parameters such as frequency and amplitude, which are crucial for damage quantification. Experiments were conducted on 3D-printed Acrylonitrile Butadiene Styrene (ABS) cantilever beams with varying crack depth ratios from 0% to 60% of the beam thickness. The study utilised the Canny edge detection technique and Fast Fourier Transform to analyse vibration behaviour captured by cameras at different settings. The results show an optimal set of camera resolutions and frame rates for accurately capturing dynamic responses. Empirical models based on four image resolutions were validated against experimental data, achieving over 98% accuracy for predicting the natural frequency and around 90% for resonance amplitude. The optimal frame rate for measuring natural frequency and amplitude was found to be 2.4 times the beam’s natural frequency. The findings provide a method for damage assessment by establishing a relationship between crack depth, beam thickness, and damping ratio.

## 1. Introduction

Dynamic response measurement is widely utilised in mechanical engineering and other engineering fields. In industries such as aerospace and manufacturing, structural components can experience issues like cracks, fatigue, deformation, and instability under operational loads. Monitoring these issues is crucial for understanding mechanical behaviour, detecting changes in mechanical properties, optimising designs, and preventing resonance-related disasters. Dynamic response measurement techniques fall into two main categories [1]: contact measurement and non-contact measurement. Contact sensors, predominantly accelerometers, have been extensively used in structural health monitoring studies over the past two decades to identify and assess structural damage and integrity [2,3,4]. Conventional contact sensors have many limitations due to wiring and additional mass [5]. Additionally, the installation and deployment of contact sensors are time-consuming and labour-intensive. The availability of space to mount the sensing elements and the complex shape pose difficulties in mounting the sensors. Those constraints can be avoided by utilising non-contact-based sensing [6,7]. In recent years, non-contact approaches have become more prevalent with advances in optics and electronics. However, while cost-effective, GPS technology is limited by its low frequency and accuracy [8,9]. GPS technology is currently only employed in a limited number of flexible structures, such as high-rise buildings and cable-supported bridges, and its usage may be limited due to systematic inaccuracies brought on by multipath and satellite constellations. By 2018, there will be a significant improvement in GPS precision while tracking dynamic displacements, according to the EU and ESA’s current Galileo plan [10].

A Doppler vibrometer (LDV) measures target vibration using the frequency change in the transmitted laser signal due to the Doppler effect. Furthermore, each LDV sensor can only measure one point of displacement, which makes it extremely uneconomical to use [11]. However, LDV devices are expensive and perform measurements sequentially, making them time-consuming and labour-intensive for large areas. In contrast, digital video cameras are cost-effective, agile, and offer simultaneous measurements with high spatial resolution [12]. A review was conducted for the evaluation of crack analysis in structures using image processing techniques [13].

Non-contact measurement methods using camera-based sensors have gained prominence. These sensors track the motions of specific targets in video images to capture structures’ dynamic responses. Unlike acceleration responses, displacement responses directly indicate structural stiffness, providing a more precise evaluation of structural conditions. Video recordings captured by industrial cameras or even smartphones can be analysed to assess and monitor structural health [14]. Several studies have shown that high-speed cameras and advanced image processing techniques effectively capture structures’ dynamic responses.

A phase-based motion magnification technique has been proposed to enhance the signal-to-noise ratio in low-amplitude measurements. This technique amplifies subtle motions in video recordings, potentially significantly improving the accuracy of dynamic response measurements in structural health monitoring. Motion magnification techniques have been applied to identify modal parameters in structures using high-speed video analysis [15]. To ensure accuracy and reliability, camera-based displacement measurements were compared with those obtained from laser vibrometers and accelerometers [16]. Motion magnification, a methodology for visualising small displacements, was extended to modal identification in structures. Laboratory experiments on a cantilever beam validated this approach against accelerometer and laser vibrometer measurements. They were used for modal analysis to visualise mode shapes and calculate mode shape curvature for damage detection [17].

Another approach for image-based vibration measurement is the Multi-Thresholding method. A video camera was implanted to capture the frequency of small signals with a low amplitude. The method traces the subpixel motions of objects in a video frame and computes the main frequency of vibration of the objects [18]. Liu and Yang [19] used neural network methods for vibration frequency prediction. In the suggested image sequence analysis, the video was read as an image sequence to target the region of interest (ROI) and saved as separate pixel brightness vibration signals. The time domain data obtained from vibration signals were then used to create frequency domain data. The computer vision tracking algorithm CSRT of OpenCV was evaluated for its ability to track vibrations. Two experiments were conducted: one with a cantilever beam and another with a robot. The resonance frequencies obtained from the vision camera method were compared to those from the industry standard laser vibrometer method [20]. Displacement monitoring uses various vision-based sensors, such as the template matching method [21] and marker tracking [22,23]. Digital image correlation (DIC) [24,25,26,27] is a widely used method for measuring displacements and strains in both 2D and 3D. In image-based methods, vibration signals are first extracted to identify vibration characteristics. Algorithms such as FFT are then used to analyze the frequency components and determine the resonant frequency and amplitude.

Patry Kot et al. [28] provided a summary of recent advancements in non-destructive testing (NDT) techniques that have greatly benefited SHM applications. These techniques, including sweep frequency, ground-penetrating radar, infrared, fibre optics, camera-based methods, laser scanners, acoustic emission, and ultrasonic techniques, have been instrumental in SHM applications. Despite challenges in data interpretation and automation, researchers are using artificial intelligence and combining NDT methods to improve accuracy. This study focuses on the latest NDT developments for monitoring concrete, masonry, timber, and steel structures.

Munawar et al. [29] presented an overview of various techniques and methodologies for image-based crack identification, concluding that crack detection approaches fall into two main categories: image processing and machine learning. Another review categorizes image-based crack detection techniques according to the type of images used in different imaging systems [30]. An overview of various image-based vibration measurement techniques and methodologies has been provided, along with details of the camera settings used [31,32,33]. The key issues investigated include accuracy, reliability, and potential challenges in implementation.

Different research studies on image-based dynamic response measurement have been applied to other materials, including steels, composites, and additively manufactured materials [34]. A greater thickness was found to increase the beam’s cross-sectional area, resulting in more interfilamentous surface friction and higher energy dissipation. This led to a higher damping ratio in test results [35].

The typical applications of high-damping structures include aerospace structures [36,37], railway structures [38], submarine structures [39], automotive parts, and other structures, where vibration management is critical. In highly damped structures, the key defects include material degradation, where damping materials lose effectiveness over time, and cracks or fractures that reduce damping efficiency. The delamination of composite layers and bonding failures can also compromise structural integrity. Additionally, the loss of damping efficiency due to ageing or environmental factors, corrosion at material interfaces, and stiffness reduction are common issues that can lead to increased vibrations and potential structural failure.

The literature shows that increased thickness and the presence of cracks enhance damping in structures. Energy dissipates through the extension and compression of the damping material under flexural stress from the base structure [40]. Damping increases with the thickness of the damping layer. The change in a material’s ability to dissipate vibrational energy directly influences its damping efficiency, impacting how well it can reduce the amplitude of vibrations in a structure. Khan et al. [41] examined the changes in dynamic behaviour due to a crack, including natural frequency, beam stiffness, and dynamic stability. They also studied crack initiation and propagation using numerical and experimental approaches. Additionally, the existence of a crack significantly increased damping, restricting the available vibration response [42,43].

One of the challenges in the damped structures is that they do not produce significant visible vibrations and, therefore, are unsuitable for capturing any measurable response through digital imaging devices for useful structural health diagnostics. This reason for their unsuitability makes researchers and industrial practitioners prefer something other than image-based dynamic response measurements for highly damped structures. The same can also be true for structures with small or hidden cracks that influence the visible vibrations almost negligibly. Despite all these valid reasons and challenges, no research is currently available to quantify the potential of digital imaging about structural damping and crack sizes that perform effective structural health monitoring.

This research quantitatively addresses the challenge of highly damped structural responses or low amplitude change due to small or hidden cracks in structures. In any image-based dynamic response measurement, the critical features of the camera are the resolution and the frame capturing speed. As is evident from the literature review, no published article has quantitatively related the potential of the mentioned features with structural dynamic response and health diagnostic capacity.

The study used additive manufacturing to effectively produce cantilever beams with varying thicknesses, resulting in different damping and vibration behaviours. Cameras recorded videos of the vibrating beams at various speeds and resolutions during the tests. Signal processing techniques and image analysis approaches were used to measure natural frequencies and resonance amplitudes from the captured videos. By analyzing the collected data, this study aims to establish trade-offs between camera settings and measurement accuracy, ultimately enhancing the reliability and effectiveness of crack detection in visual-based vibration monitoring.

## 2. Materials and Methods

This study used ABS filament manufactured by Ultimaker^®^ (Utrecht, The Netherlands). Red ABS, one of the most widely used FDM polymers, was employed as a raw material due to its ability to demonstrate vibration behaviour and allow researchers to precisely observe small cracks and plastic zones [44]. The excellent mechanical properties of ABS have made it a popular research choice for various industrial applications [45]. ABS is lightweight and is useful in situations where low weight is essential, for instance, in the aerospace and automotive industries, and its high-impact strength makes it less likely to break under stress [46]. It is also resistant to many chemicals, making it suitable for use when it comes into contact with corrosive substances [47,48]. ABS filament with a diameter of 2.85 mm was used. The ABS employed was in the shape of a filament that would be extruded using the heated extremities of the 3D printer nozzle by inserting the necessary geometric specifications into the 3D printer [49]. Sample components were manufactured using variable 3D parameters of varying thicknesses, which created different dynamic response parameters. Additional information regarding fused deposition modelling (FDM) ABS can be found in Table 1.

### 2.1. Specimen Geometry

**Figure 1:**
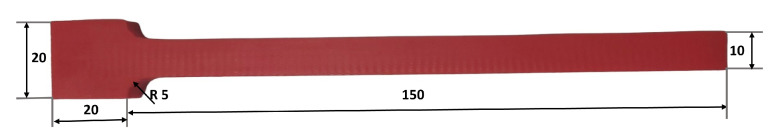
One specimen was printed using a nozzle size of 0.4 mm, 0° raster angle printing orientation, and 0.5 mm layer thickness (Unit: mm).

The specimen’s geometric layout was created using the CATIA v5 CAD software. A cantilever beam structure was used in the study to examine the dynamic characteristics of the fabricated specimens. The cantilever beam method was seen to be ideal for fixing the beam at one end to measure its vibrational attributes from the other end [51]. Figure 1 illustrates the chosen dimensions of the ABS specimen. The geometry of the beam structure is 150 mm long with 10 mm in width. The study included a set of specimen dimensions carefully designed to explore a range of thicknesses. These specimens were of varying thickness, extending from a minimal 3 mm to 6 mm, with a consistent stepped interval of 1 mm between each successive thickness. The final design of the specimen CAD model file, saved in Stereolithography (STL) format, was exported as G-code files for printing. This process was made possible by the user-friendly Ultimaker Cura 4.4 software, which can adapt to various 3D printing needs. The design was then printed using the reliable Ultimaker 2+ 3D printer.

### 2.2. 3D Printing Parameters

**Figure 2:**
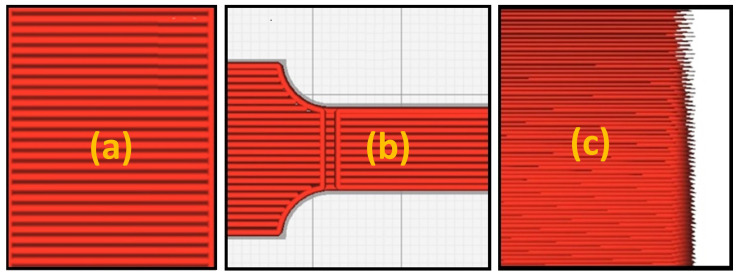
Printing Parameters: (**a**) Nozzle sizes = 0.4 mm. (**b**) Printing orientations = 0°. (**c**) Layer thickness = 0.05 mm.

Table 2 shows four significant printing parameters (printing orientation, nozzle size, infill density, and layer thickness) that were taken into account in this research at various beam geometrical dimensions, because prior research proposed that these parameters had the most significant effect on structural damping behaviour [35]. Various beam thicknesses were used, and the 3D printing parameters were adjusted to create components with varying damping structures. These parameters also impacted the FDM process’s other dimensions, including the manufacturing duration and expenses entailed. According to [52], a printing orientation of 0° had the most significant impact on damping behaviour. The infill density has the most significant influence on damping behaviour [35]. Furthermore, the nozzle size of 0.4, mm fit in terms of printing time and increased the damping in specimens. The layer thickness was determined using default configurations. These settings represented the standard setting range of the chosen 3D printer and produced outstanding printing quality. Figure 2 depicts the chosen values given for each parameter in Table 2.

In the case of a beam, increasing the thickness led to an increase in damping. The reason is that thicker beams typically have a larger cross-sectional area, which increases internal friction and energy dissipation.

### 2.3. Experimental Scheme

Various specimen thicknesses were used to generate different vibration characteristics. These beams were 3 mm to 6 mm thick, with a consistent 1 mm interval between each sample. Consequently, the research included four specimens of different thicknesses, which were all subjected to complete impact and resonance testing analyses. These different specimen thicknesses were employed with the aim to generate different levels of structural damping in beams by varying beam thicknesses, incorporating 3D parameters and crack depth. The proposed crack depth ratio was based on the beam thickness [53,54] and was located on the top surface of the beam to analyse its effect on the dynamic response closer to the fixed end of the beam. The crack depth ratio was 5 mm away from the beam’s fixed end. To analyse their effect on the dynamic response, crack depth ratios of 0%, 20%, 40%, and 60% concerning the thickness of the specimen were suggested in this study to evaluate the beam structure. These cracks were used to investigate the behaviour of a cracked beam to quantify damages [55,56,57,58,59,60]. The requirement for high-speed cameras in high-vibration environments is a significant challenge, primarily due to the associated costs. The costs of high-speed cameras require accurate frame rate and high resolution, which can increase the cost of these cameras. Moreover, the high frame rates quickly generate large volumes of data, necessitating high-capacity storage solutions and fast data transfer systems, which add to the overall cost.

During the resonance testing phase, the camera’s frames per second (FPS) settings were adjusted to capture videos of the beam’s oscillations. The varying beam thicknesses present a challenge in selecting the FPS as constants to examine the dynamic response of beam structures, given their different natural frequencies. The approach was used to select the FPS based on the natural frequency of each beam thickness. The FPS values were selected within a range of 1.8×f, 2×f, 2.2×f, and 2.4×f, where the variable *f* represents the fundamental frequency. Here, it was determined using an accelerometer during the impact test. The camera resolutions were set to four constant values, 800×600, 960×720, 1120×840, and 1280×960 pixels, since they represent commonly used cameras’ values. The complete experimental design was used to comprehensively evaluate the influence of the camera setups on the measured dynamic properties in presenting small cracks in damped structures. Table 3 shows the experimental scheme of the dynamic response tests, where is n is several-fold of f fundamental frequency.

## 3. Experimental Setup and Procedures

**Figure 3:**
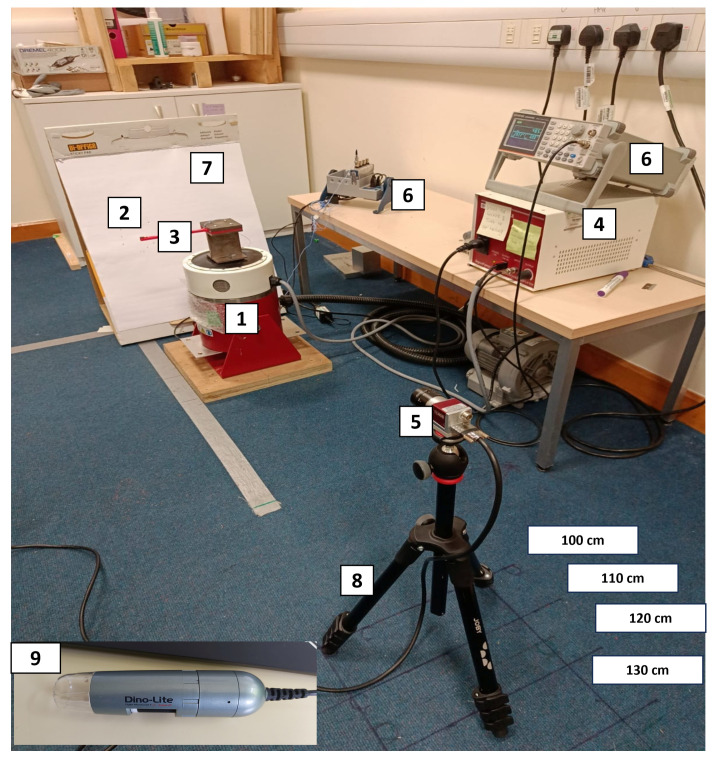
Experimental setup and procedures. ^1^ Shaker ^2^ Accelerometer ^3^ Specimen ^4^ Power Amplifier ^5^ Camera ^6^ Function Generator ^7^ White Background ^8^ Tripod ^9^ Microscopy Camera.

This study investigated the cantilever beam structure under sudden and resonance frequency force. This experiment used impact and resonance tests on a beam structure to measure the fundamental frequency, damping ratio, and resonance amplitude. The resonance frequency of the beam structure was examined to assess how the camera setup influences the image-based dynamic response measurement in different damping structures. Figure 3 shows the complete experimental setup.

A set of equipment and software was successfully integrated into the experimental vibration setup. The signal generator AFG21105 generated a precise sinusoidal output. A dedicated power amplifier then magnified this signal to create the force to induce vibration in the shaker. The fixed end of the ABS beam was mounted on the shaker. The beam was excited by the motion obtained from the shaker with the constant displacement amplitude. An accelerometer PCB 352A21 was fixed into the beam’s free end, allowing for the measurement of time and acceleration data at a 25,600 Hz sampling rate. The data were captured using an accelerometer and imported into the SignalExpress 2015 software through the NI 9174 DAQ chassis equipped with a DAQ card. The SignalExpress 2015 software visualised real-time data and saved it as a “.txt” file format for subsequent analysis. The text files were then exported to MATLAB to analyse and process the beam structure’s dynamic response. The beam structure’s dynamic response was measured using the same steps and resonance tests. The difference is that the impact test was conducted three times to determine the beam’s fundamental natural frequency and damping ratio by averaging them. The resonance was used as the natural frequency obtained from the impact test. It was fed into the signal generator to drive the shaker, producing sinusoidal vibrations with resonance frequency to measure the maximum amplitude. The crack depth was captured using a Dino-Lite Pro AM3613TB microscopy camera, and the crack depth was measured using the Dino Capture 2.0 software. Meanwhile, an AOS PROMON U750 camera (manufacturer: AOS Technologies AG, Tring, United Kingdom) was fixed on a tripod positioned at a distance of 100 cm from the beam structure. It was linked to the laptop’s USB port, allowing the recorded videos to be stored using AOS Imaging Studio V4 software. The camera software was used to save the test’s captured video in “.raw4” file format; it then exported the videos in “.avi” format or a set of images with suitable format for more analysis. The camera software provides options for configuring essential parameters to capture the dynamic response as the user desires. The camera’s exposure time, white balance, and other settings were fixed throughout the experiment. The purpose of this recording was to gather data to extract dynamic features for further analysis using MATLAB with image and signal processing.

### 3.1. Experimental Flow Diagram

**Figure 4:**
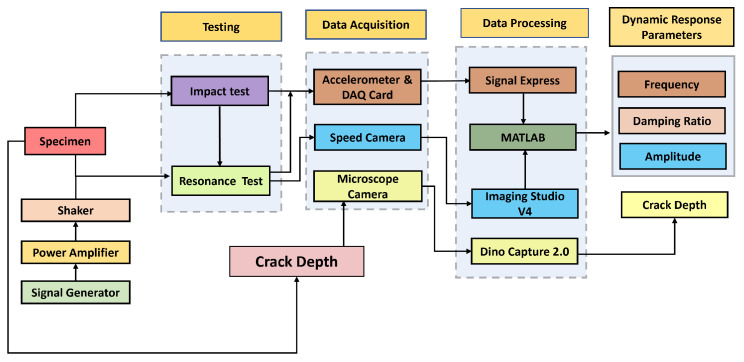
Experimental flow diagram.

Figure 4 presents a systematic flow diagram of the experimental procedures. These procedures were designed to collect data on dynamic response parameters, employing a diverse range of data collection methods. The flow diagram was divided into types of testing used to collect the data using different acquisition systems, such as cameras and an accelerometer, as well as data processing software to analyse the data obtained and other devices used to perform the testing.

#### 3.1.1. Impact Test

**Figure 5:**
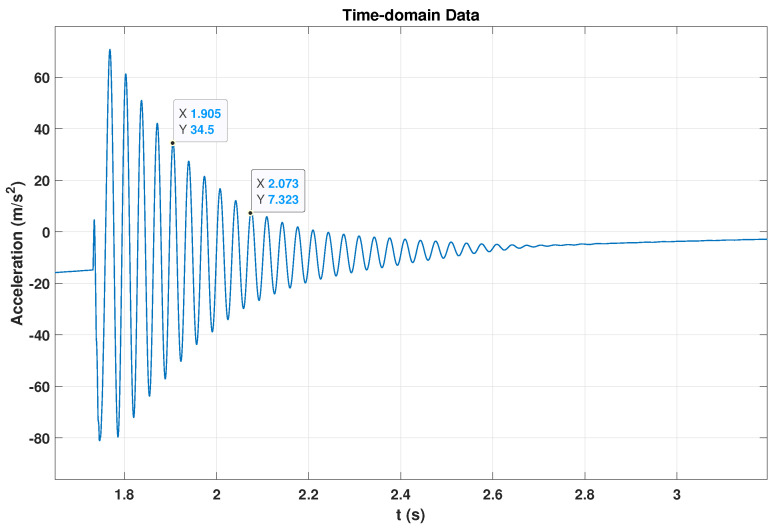
Time domain data.

Impact testing is a fundamental technique within an experimental approach analysis. It enables the determination of a structure’s natural vibration frequencies. This project used the impact test to evaluate the behaviour of the ABS cantilever beam. In this test, a sudden force was applied to the free end of the ABS beam by striking it with a finger or a small tool. The accelerometer was fixed to the same free end to record the acceleration over time at a sampling rate of 25,600 Hz. The recorded data were imported into the MATLAB application and plotted in the time domain to measure the fundamental frequency and damping ratio. The fundamental frequency determines the difference in indices and corresponding time values of the two peaks, as shown in Figure 5 the time (t) values in the x axis for 1.905 s and 2.073 s.

#### 3.1.2. Damping Test

**Figure 6:**
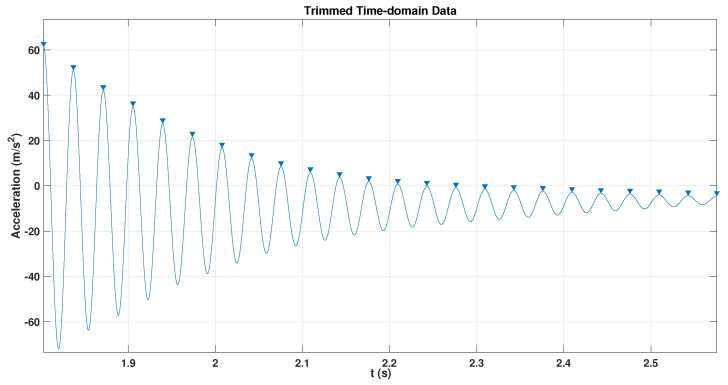
The gradual reduction in the amplitude of free vibrations.

Damping tests are essential in various engineering fields, including civil, mechanical, and aerospace engineering. Various techniques are generally used to measure damping capacity. The logarithmic decrement method, also called the free decay method, represents the most straightforward approach for determining damping in a beam. In this technique, damping characteristics are derived by assessing the gradual reduction in the amplitude of free vibrations, as illustrated in Figure 6. Subsequently, the logarithmic decrement is calculated between every two peaks and the resulting values [61]. It is commonly applied when performing modal damping for structural vibrations [62,63,64]. The damping test involved subjecting the cantilever beam to three sudden force excitations to initiate free vibration across all thickness variations. Then, the average of the damping ratio measurements was calculated.

#### 3.1.3. Resonance Test

**Figure 7:**
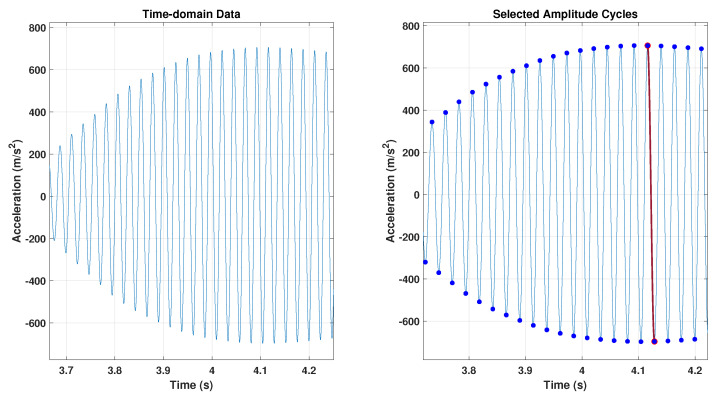
The resonance amplitude.

Y.B.Yang et al. [65] provided a study that recently investigated the importance of beam resonance for a better understanding and examined resonance and cancellation conditions in beams with moving loads. Resonance testing is a method that uses the natural vibration frequencies of a structure or a system to measure resonance amplitude. Impact tests measured the fundamental frequency of the intact and cracked beam. Then, the beam was excited under the resonance vibration to measure the maximum amplitude. The maximum amplitudes were determined by measuring acceleration values and time intervals at peak and trough points. The top point in the red line is the peak point, while the bottom is the trough point, as shown in Figure 7. Both an accelerometer and a camera were used during the resonance to collect the data for further analysis to measure the resonance amplitude.

## 4. Data Processing and Analysis

Raw data were obtained from the accelerometer and camera. The data recorded by the accelerometer were processed using SignalExpress 2015. Meanwhile, the speed camera captured test videos using its application. The accelerometer’s time, acceleration data, and test videos were imported into MATLAB R2019b for analysis.

### 4.1. Accelerometer Data Analysis

The accelerometer data were initially captured using SignalExpress 2015 software and then saved as a “.txt” file format. These text files, containing both time and acceleration data, were then imported into MATLAB for additional analysis. Within MATLAB, the beam’s fundamental frequency (*f*) was measured using Equation (1). Herein, $t_{i}$ and $t_{j}$ were employed to represent the time of occurrence of the *i*th and *j*th peak amplitude, respectively.

(1) $$f=\frac{i-j}{t_{i}-t_{j}}.$$

Equation (2) was employed to compute the maximum amplitude of the first-order resonance. This particular amplitude denotes the utmost displacement reached by the system throughout the resonance test. The variable symbolizes the greatest amplitude of acceleration that occurred during the resonance test. Thus, we have the following:

(2) $$U=\frac{a_{\max}}{{\left(2\pi f\right)}^{2}},$$

where *U* denotes the displacement amplitude, and $a_{\max}$ denotes the acceleration amplitude. The damping ratio ζ is defined in Equation (3), which relies on the natural logarithm of the peak acceleration ratio, while the $a_{i}$ and $a_{j}$ correspond to the accelerations of the *i*th and *j*th peaks, respectively.

(3) $$\zeta =\frac{\ln\left(\frac{a_{i}}{a_{j}}\right)}{\sqrt{{\left[\ln\left(\frac{a_{i}}{a_{j}}\right)\right]}^{2}}{+[\left(2\pi \left(j-i\right)\right]}^{2}}.$$

### 4.2. Camera Data Analysis

The experiment videos of the vibrating beams were captured and converted into a sequence of images for each video and then used in MATLAB for processing. The tip coordinates of the beam were determined and recorded in each frame using MATLAB script code. These coordinates were displayed in the time domain based on the camera speed rate. These data were then converted to the frequency domain using FFT to determine the dynamic response characteristics.

#### 4.2.1. Image Assessment

**Figure 8:**
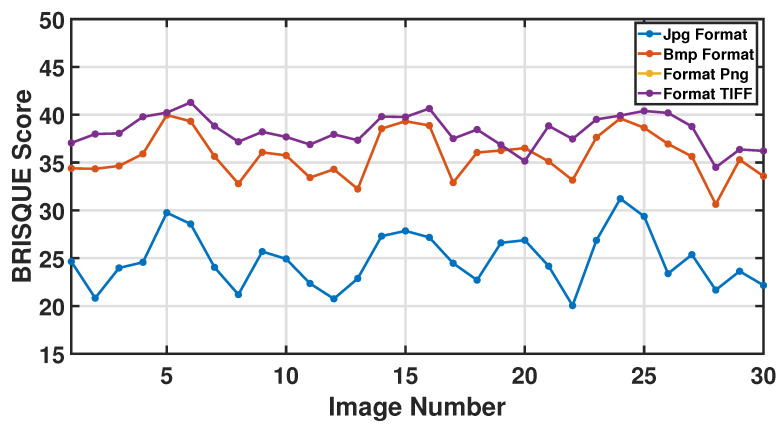
BRISQUE scores for 30 images with different image formats.

Incorporating the No-Reference Image Quality Assessment (NR−IQA) through the The Blind/References Image Spatial Quality Evaluator (BRISQUE) methodology is integral to our approach to determining the optimal image format. Figure 8 shows the result of 30 BRISQUE scores listed across different image formats: JPG, BMP, PNG, and TIFF. These scores measure image quality, with lower scores indicating better quality. BRISQUE measures the number of artefacts and distortions in an image, so a lower score suggests fewer artefacts and distortions and, therefore, better quality. Generally, JPG images have lower BRISQUE scores, recommending better image quality for most images. BMP images have relatively higher BRISQUE scores than JPG, and BMP is a lossless format but can result in larger file sizes. PNG scores vary but are mostly lower than BMP and higher than JPG, and PNG is a lossless format that supports transparency and is often used for images with sharp edges, text, or areas of uniform colour. If the goal is to minimise artefacts and distortions, especially in images with smooth colour transitions, JPG is the best-performing format in this specific set of images. However, choosing the ‘best’ format depends on the images’ particular characteristics and the application’s requirements. Ultimately, the choice of image format depends on the user’s specific requirements, such as whether the user desires a smaller file size, lossless compression, or other factors.

#### 4.2.2. Beam Tips Detection

An AOS PROMON U750 camera was mounted on a tripod located 100 cm from the beam, which was fixed on a shaker and linked to a laptop using the USB port. It was used to record the resonance vibration of the beam. The camera recorded the tests with the help of Imaging Studio V4 software for later analysis. The experimental scheme included four of the beam thicknesses. By selecting one beam thickness, 64 possible combinations can be formed.

**Figure 9:**
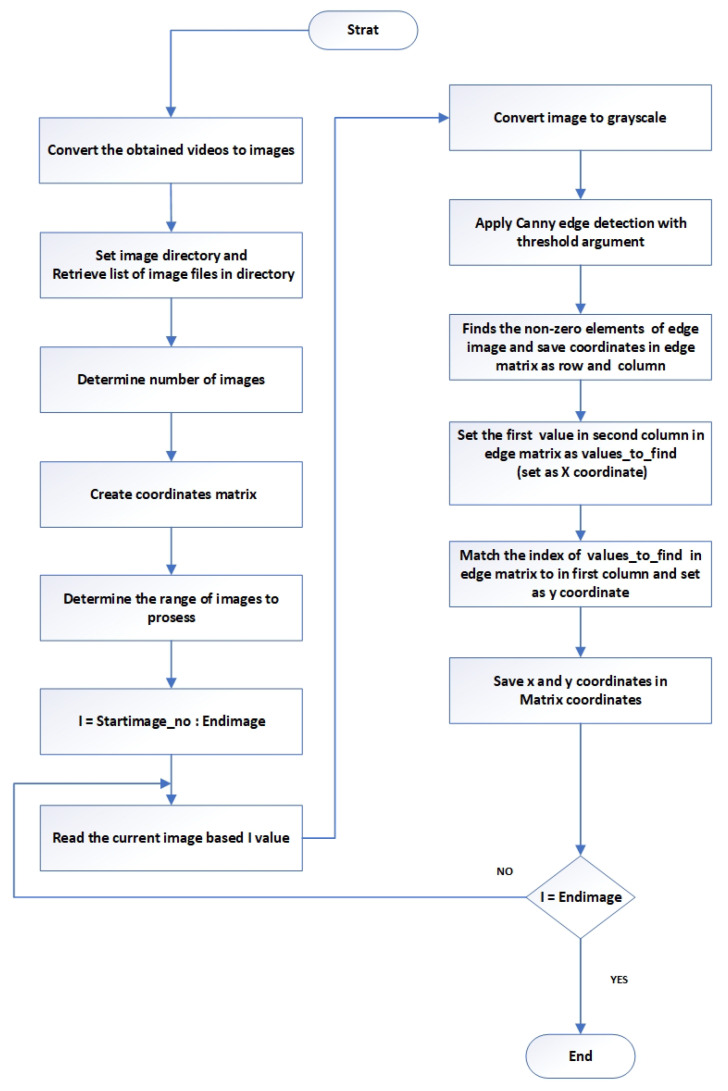
Beam tip detection flowchart.

The fundamental frequency and resonance amplitude were extracted from the camera data using the following method: first, the resonance testing was recorded using a speed camera to capture the movement of the beam with different camera settings, as proposed. The next step involved analysing the data to measure the dynamic response of the structure’s (beam) vibration. This was done by applying appropriate image processing techniques to detect the tips of the vibrating beam, as illustrated in Figure 9. The fundamental frequency and resonance amplitude were carefully extracted from the camera data using the following method: first, the resonance testing was recorded with the utmost precision using a speed camera to capture the movement of the beam with different camera settings, as proposed. The next step involved thoroughly analysing the data to measure the dynamic response of the structure’s (beam) vibration. This was done by applying appropriate image processing techniques to detect the tips of the vibrating beam.

In the initial phase of the process, an approach was adopted to convert the experiment videos into sequences of images. This involved setting up the image directory to retrieve the list of image files in this directory. At this point, the number of images in this directory was counted. An empty matrix was created to store the tip’s coordinates *x* and *y* for each image count based on the user’s input. The range of images to be processed (starting and ending images) was determined. The initial image of the selected ones was read and converted to grayscale.

Grayscale conversion is a common preprocessing step for detecting edges. The grayscale image went through the Canny Edge detection algorithm. This algorithm helps find the edges or boundaries of objects in an image. The threshold parameter controls edge detection sensitivity and can be adjusted.

The coordinates of non-zero elements in the edge image should be saved in the edge matrix as rows and columns. In the edge matrix, the first value in the second column is the *X* Coordinate of the beam’s tip (values to find). Then, the index of the values was matched to find in the edge matrix with the first column and set as the *Y* Coordinate. Their coordinates were extracted from the recorded data after detecting the beam tips.

**Figure 10:**
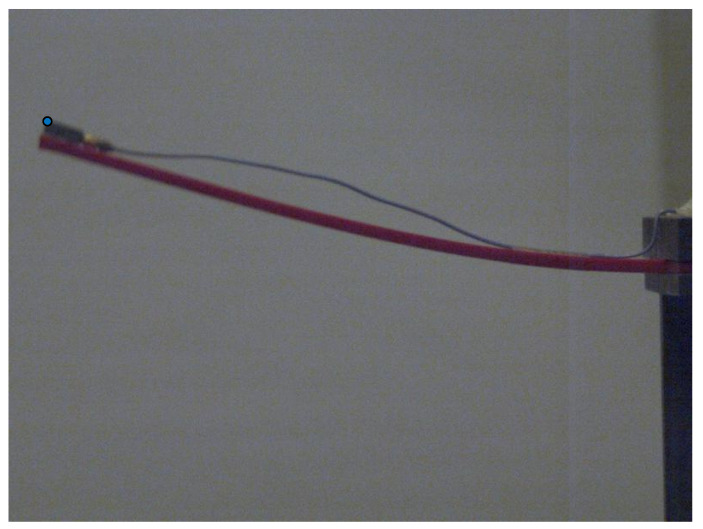
Beam tip detection in coloured image.

**Figure 11:**
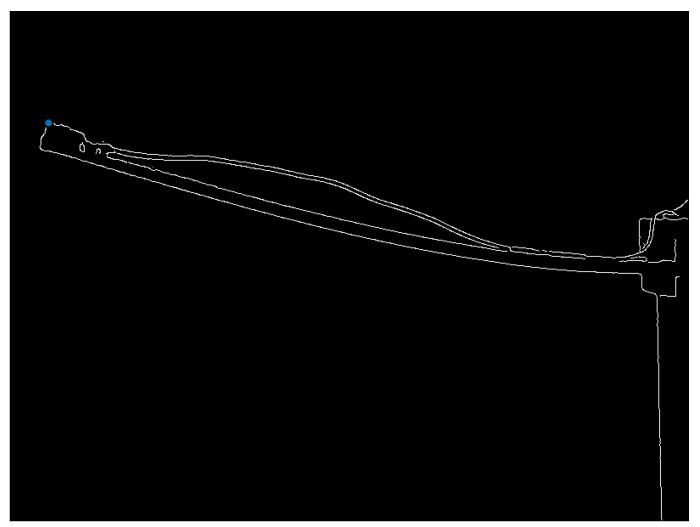
Beam tip detection in the edge image.

Figure 10 displays the original image’s beam tip. In Figure 11, the x and y positions of the detected beam’s tip are represented by blue dots in the edge detection image. These coordinates are stored in a matrix. After saving the file, the user can open it in MATLAB for additional processing and analysis.

### 4.3. Camera Time Domain Analysis

**Figure 12:**
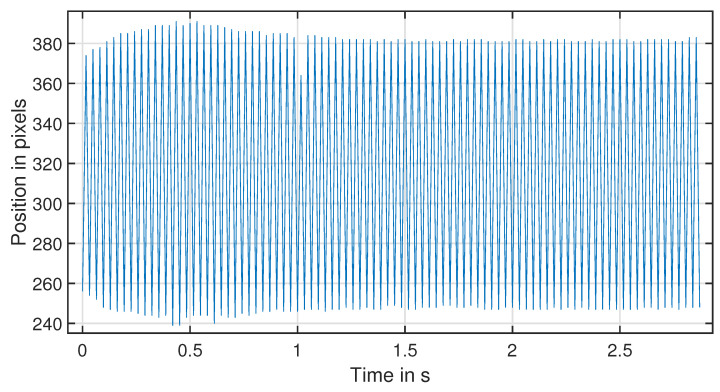
The camera time domain analysis.

One way to perform time domain analysis is to use a camera to track the beam tip position over time in each frame. The sampling rate of this process depends on the frame rate of the video recording and the number of samples per frame. A time vector can be created by dividing the number of frames by the sampling rate and the number of samples per frame. If the test video duration is 2 s and the video is recorded at 50 frames per second, the time vector can be calculated in a logical sequence. First, the time interval was determined as 1/50=0.02. Then, the number of images was calculated as 2×50=100. Therefore, a time vector that spans from 0 to 1.98 s in increments of 0.02 s was determined to be needed to record 100 images in 2 s. The time vector = [0.0, 0.02, 0.04, 0.06, 0.08, 0.1,…, 1.98]. The *y* position of the beam’s tip can be plotted against the time vector to show the beam tip’s motion over time. The time domain of the vibration beam structure is shown in Figure 12.

### 4.4. Camera Frequency Domain Using FFT

**Figure 13:**
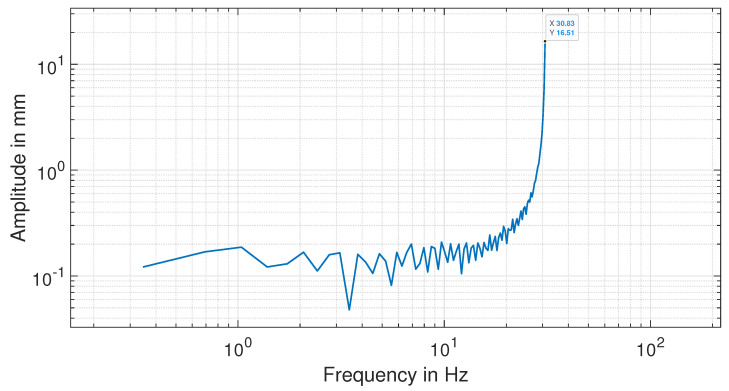
Camera frequency domain using FFT.

Figure 13 shows the result of using FFT to convert the beam vibration image-based time domain to the frequency domain. This technique transformed the time domain data into the frequency domain, where dynamic response parameters measure the resonance amplitude and the fundamental frequency. The beam’s tip position data were also subjected to Fourier analysis, revealing the signal’s essential frequencies. In the time domain, the y position was plotted against time in seconds in pixel units, which needed to be scaled to mm. Finally, graphs were drawn to display the data. The analysis of vibrations relies on two key parameters: the resonance amplitude and the fundamental frequency. The *X* axis is Frequency in Hz, whereas the *Y* axis is amplitude in mm. It can be observed that the *y* axis had an amplitude of 16.51 mm at a frequency of 30.83 Hz. This frequency is assumed to be the resonance frequency of this cantilever beam.

## 5. Experimental Results

Data from the accelerometer and high-speed camera were collected. The crack depths were measured using a Dino-Lite digital microscope camera, and the maximum amplitude of the beams was measured using a resonance test. The fundamental natural frequencies and damping ratios were calculated using an impact test. The discussion will concentrate on the influence of the dynamic response parameters on other experimental variables. The fundamental trends and correlations will be analysed and presented.

### 5.1. The Dynamic Response of a Cantilever Beam Undergoing Free Vibration

The dynamic response of a free-vibrating cantilever beam may be analysed in terms of the beam’s maximum displacement occurring at the free end of natural frequencies. When a cantilever beam is subjected to free vibration, it vibrates without any external forces acting on it after an initial disturbance. The natural frequency, with the largest amplitude, is the most significant in many practical situations.

#### 5.1.1. Fundamental Frequencies for Intact and Cracked ABS Beam as Measured by Accelerometer

**Figure 14:**
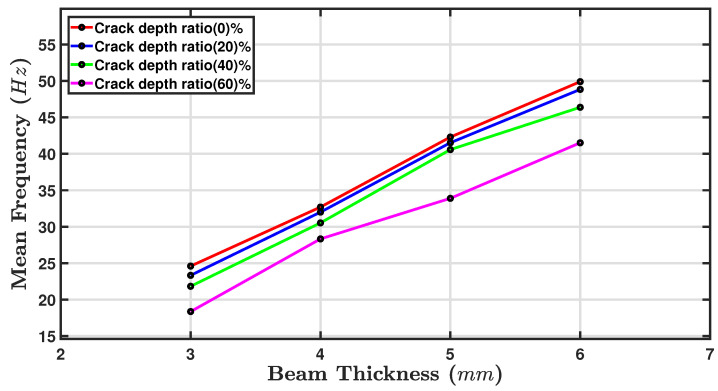
The relationship between mean frequency and beam thickness.

The fundamental frequencies of a beam can be significantly affected by the presence of a crack. The crack changes the local stiffness of the ABS beam, which in turn influences the vibration behaviour [66,67]. Of course, increasing the thickness of a beam directly increases its stiffness, which increases its natural frequencies. Figure 14 shows a line graph of the mean frequency (Hz) plotted against beam thickness in mm. The four lines represent different crack depth ratios (depth of crack/beam thickness), which are expressed as percentages: 0, 20, 40, and 60%. The beam thickness was from 3 mm to 6 mm in increments of one mm. The mean fundamental frequency ranged from approximately 18 Hz to 55 Hz. Figure 14 shows that as the beam thickness increased, the mean of the first or fundamental frequency [68] also increased for a given crack depth ratio. This trend was consistent across all thicknesses and crack depths, showing a linearly positive correlation between beam thickness and mean frequency: For a given beam thickness, as the crack for a given beam thickness and as the crack depth ratio increased, the mean frequency decreased [69], as shown. For instance, at a 3 mm beam thickness, the mean frequency was approximately 24 Hz for zero crack depth, while for a ratio of 60%, it was around 18 Hz. The mean frequency for beams with no cracks (0% crack depth ratio) was consistently the highest for all thicknesses, and the greatest crack depth ratio (60%) always resulted in the lowest mean frequency.

#### 5.1.2. The Influence of the Crack Depth on Natural Frequencies

**Figure 15:**
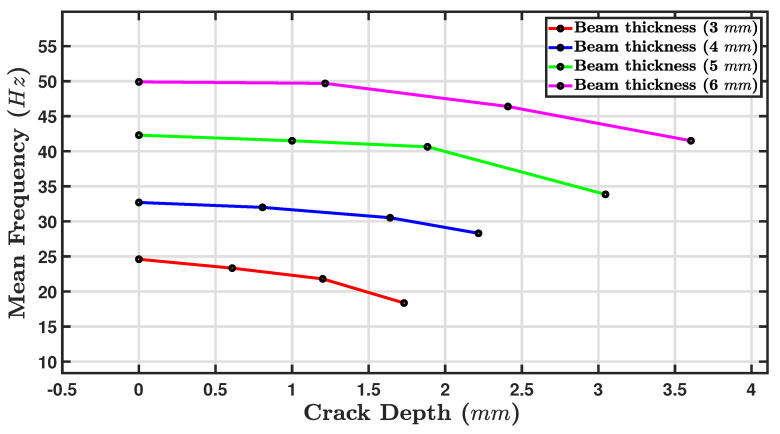
The relationship between mean frequency and crack depth.

Experimental and analytical findings from various research papers confirm that as cracks developed in beams, the structure’s natural frequency decreased; as a crack developed, the natural frequency of the cracked beam reduced [57,70,71,72]. Figure 15 shows a line graph that illustrates the relationship between mean frequency (Hz) and crack depth (mm). The four lines on the graph each correspond to a different beam thickness: 3 mm (red), 4 mm (blue), 5 mm (green), and 6 mm (magenta). The crack depth ratio ranged from 0 mm to just over 4 mm. The mean frequency ranged from approximately 18 Hz to 49 Hz. It can be clearly seen that for all four beam thicknesses, as the crack depth increased, the mean frequency decreased due to the decrease in stiffness of the beam [73,74]. This inverse relationship indicates that the presence of a crack in the material of the beam decreases the beam’s fundamental frequency. The steeper the slope of the line, the more pronounced the effect. The mean fundamental frequency of the 3 mm thick intact beam (red line) was about 24 Hz, but for the same beam with a 1.7 mm deep crack, the mean fundamental frequency decreased to about 20 Hz. The intact beam of 6 mm thickness (magenta line) had a mean fundamental frequency of approximately 49 Hz. However, the introduction of a crack of the depth of 3.6 mm decreased it to around 41 Hz. However, the rate of decrease in the mean frequency as the crack depth increased was steeper for thicker beams. For instance, the 6 mm beam showed a more gradual decline in mean frequency compared to the 3 mm beam over the same crack depth range.

#### 5.1.3. Investigate the Damping Characteristics of the FDM ABS Cantilever Beam

The damping ratio is an essential concept in engineering that measures the rate at which the amplitudes of the vibrations in a structural system decrease. It is usually represented as a ratio of two values: the actual and critical damping coefficients. Many factors influence the damping ratio of a vibrating beam: not only the properties of the beam, but even the type and position of the accelerometer used for measurement. Other important factors will be the process parameters used in the 3D printing (material extrusion) of the ABS beam, especially its geometry and material properties. One reason for different damping ratios when using beams of different thicknesses and crack depths may be due to the nozzle size used for the FDM, since it has been found that the damping ratio is higher when using smaller nozzles [75]. Greater beam thickness means increased cross-sectional area, so there is more interfilament surface friction, causing higher energy dissipation and leading to increased damping in the beam [35].

The changes in the damping ratio as the crack depth increased from 0% to 60% are shown in Table 4, and Table 5 presents the mean fundamental frequency $F_{mean}$ of an ABS polymer cantilever beam for various beam thicknesses and crack depth percentages $C_{d}$.

Table 4 shows fluctuations in the damping ratio as the crack depth increased from 0% to 60%. At 0%, 20% and 40% crack depth ratios, the 5 mm thick beam showed the highest damping ratios, but there were some deviations from the guideline that the thicker the beam, the greater the damping. At the zero crack depth, the 6 mm thick beam exhibited the greatest damping. The 60% crack depth ratio is interesting, because the highest damping ratio, 0.0130, occurred for the thinnest beam of 3 mm.

In Table 5, showing the $F_{mean}$ against beam thickness for four values of $C_{d}$, two clear trends can be observed: (i) the thicker the beam, the higher the value of $F_{mean}$, and (ii) the greater the crack depth ratio, the lower the value of $F_{mean}$. Thus, the highest observed fundamental frequency was for a beam thickness value of 6 mm and a zero crack depth of 49.87 Hz, while the lowest observed value of the $F_{mean}$ was 18.71 Hz for a 3 mm beam with 60% $C_{d}$. The trade-off between damping and fundamental frequency will depend on the specific application requirements.

The fundamental frequency represents the natural vibration frequency of the beam. As the crack depth increases, the fundamental frequency tends to decrease. The key findings are shown in Table 5: The highest fundamental frequency observed for a beam thickness of 6 mm came out to 49.87 Hz, because the stiffer beam (greater thickness) resulted in higher natural frequencies. At the 20% crack depth ratio, the highest fundamental frequency for a beam thickness of 6 mm was 49.71 Hz. The 40% and 60% crack depth cases showed a consistent decrease in their fundamental frequency values with increasing crack depth.

Increasing beam thickness generally enhances damping but may affect the fundamental frequency. The trade-off between damping and fundamental frequency depends on the specific application requirements.

Mean Fundamental Frequency Fmean for different crack depth ratios and thicknesses of ABS cantilever beam.

#### 5.1.4. The Influence of Beam Thickness on Damping Ratio

The thickness of a cantilever beam generally leads to increased damping. A thicker beam’s higher moment of inertia increases its stiffness and resistance to bending. As a result, the energy dissipated through damping mechanisms, such as material internal friction or air resistance, is higher, reducing the amplitude of vibration.

**Figure 16:**
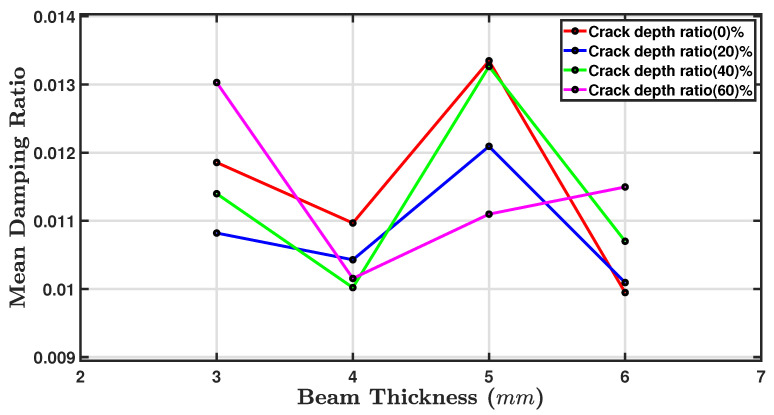
The relationship between mean damping ratio and beam thickness in different crack depth ratios.

Figure 16 displays the relationship between the mean damping ratio on the y axis against the beam thickness in millimetres (mm) on the x axis. The chart contains four markers, with each represented by a different colour and corresponding to a different crack depth ratio. The mean damping ratio generally increased as the beam thickness decreased for all crack depth ratios. However, there was a different pattern where the damping ratio decreased as the beam thickness rose from 3 mm to 4 mm, then it sharply increased at 5 mm and consequently decreased again towards 6 mm. This pattern was consistent across all crack depth ratios but was most notable for the 0% and 20% crack depth ratios. The markers for the 40% and 60% mean damping ratios were closer, pointing to the implication that the mean damping ratio is more consistent across different beam thicknesses when the crack depth ratio is higher. It was observed that the highest average damping ratio was achieved at a beam thickness of 5 mm, regardless of the crack depth ratio. The results indicate that a thickness of 5 mm provides the best damping performance for the given crack depths. On the other hand, the lowest average damping ratios were found at 3 mm and 6 mm thicknesses for all crack depth ratios. The beam’s sensitivity to damping is such that even the slightest change in position can significantly alter the measured damping. The role of boundary conditions in the experiments cannot be overstated. They significantly influence the damping values obtained, while the natural frequencies remain relatively stable [76].

### 5.2. The Dynamic Response of a Cantilever Beam under Resonance Frequency Measured by Accelerometer

The influences of beam thickness and crack depth on the resonance amplitude of the beam structure under an applied force acting at the resonance frequency force measured using an accelerometer are reported and discussed.

#### 5.2.1. The Influence of the Beam Thicknesses on the Resonance Amplitude

**Figure 17:**
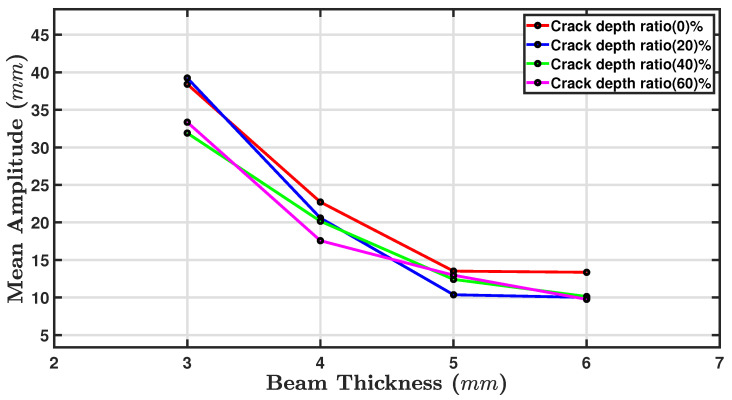
Mean amplitude as a function of beam thickness for four crack depth ratios.

Figure 17 shows the mean amplitude as a function of beam thickness for four crack depth ratios. As the beam thickness increased, the mean amplitude consistently decreased for all crack depth ratios [73,77], suggesting that the amplitude of the response is inversely related to the thickness of the beam, regardless of the crack depth ratio. The line representing the 0% crack depth ratio started with the highest mean amplitude at the thinnest beam thickness, 3 mm, and it ended with the lowest amplitude for the thickest beam of 6 mm. Likewise, the lines for the 20%, 40%, and 60% crack depth ratios also generally showed a decrease in their mean amplitudes as the beam thickness increased, with the 60% crack depth ratio generally having the lowest mean amplitude values. The graph confirms that the presence of cracks affects the mean amplitude with greater crack depth ratios, producing a lower mean amplitude for any given beam thickness. This indicates that cracks significantly dampen the mean amplitude [78].

#### 5.2.2. The Influence of Crack Depth on the Resonance Amplitude

**Figure 18:**
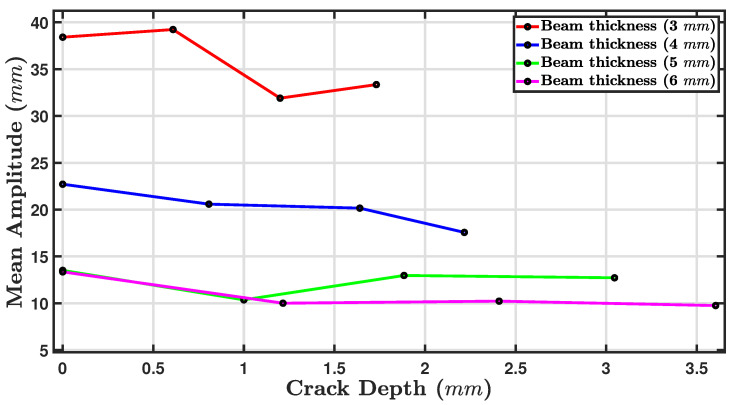
The relationship between mean amplitude and beam thickness.

Figure 18 shows a plot of the mean amplitude (mm) against the crack depth (mm) for four thicknesses: 3 mm (red line), 4 mm (blue line), 5 mm (green line), and 6 mm (magenta line). The mean amplitude decreased as the crack depth increased for the beams with thicknesses of 3, 4, and 5 mm. This trend was most notable for the thinnest beam of 3 mm, where the mean amplitude started near 40 mm for the zero crack depth and fell to just above 35 mm for a crack depth of 3.5 mm [79]. The 4 mm and 6 mm beams showed a more gradual decline in amplitude with increasing crack depth. For the beam with a thickness of 5 mm, the mean amplitude first dropped and then rose to around 13 mm, after which it remained approximately constant. It appears that the thicker beams are less sensitive to the effects of the crack depth on the mean amplitude than thinner beams. The relationship between the crack depth and vibration amplitude is complex and relies on a number of considerations, including, as stated above, the location of the crack, the material properties, and the geometry of the beam. Previous research has indicated that the vibration amplitude decreases with crack depth [45,59,67]. Because of increased damping, the energy dissipated during vibration increases, leading to reduced displacement amplitude.

### 5.3. The Dynamic Response of a Cantilever Beam under Resonance Frequency as Measured by Camera

#### 5.3.1. Influence of Camera Speed on Measured Natural Frequency

**Figure 19:**
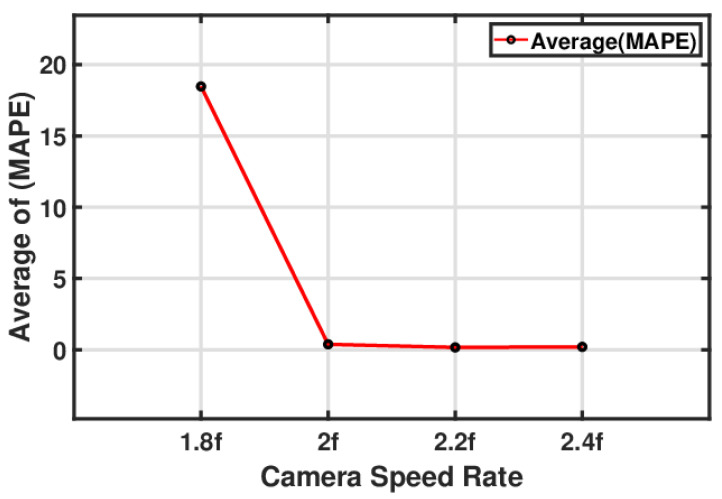
Average mean absolute percentage error for fundamental natural frequency as a function of camera speed.

Camera speed rates, data window size, and data sampling rates can significantly impact the accuracy of capturing the magnitudes and frequencies of natural beam vibrations [80]. Figure 19 shows a line graph by which to assess the camera’s speed and image-based measurement of the natural beam vibrations. The mean absolute percentage error (MAPE) for the resonance amplitude was used to calculate the error percentage. The camera speed rate is shown as multiples of ‘*f*’, where ‘*f*’ represents the fundamental natural frequency, with values ranging from 1.8 *f* to 2.4 *f*. The graph reveals a significant reduction in MAPE when the camera speed rate reached 2 *f*. This reduction rapidly decreased the error to almost zero. The sharp drop indicates that it is critical to have a camera speed rate at least double that of the natural frequency (as per the Nyquist–Shannon sampling theorem) to achieve accurate measurements. As the camera speed rate increased from 2 *f* to 2.4 *f*, the MAPE improved significantly and stabilised at zero beyond a specific speed rate.

#### 5.3.2. Influence of Camera Speed on Measured Resonance Amplitude

**Figure 20:**
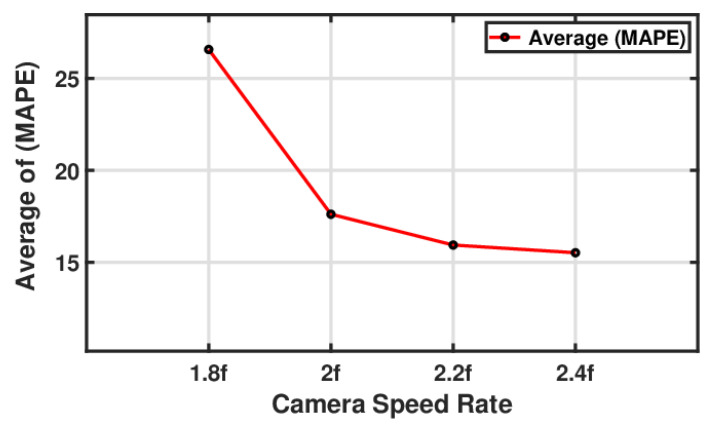
Average mean absolute percentage error for resonance amplitude as a function of camera speed.

Figure 20 shows a line plot that assesses the camera frame rate by identifying the smallest error percentage of the image-based resonance amplitude measurement of the beam structure. The line, representing the average of the MAPE, connects several data points at different camera speed rates. On the left side of the graph, the MAPE is highest at approximately 26% for a camera speed rate of 1.8 *f*. As the speed rate increased, the MAPE decreased, reaching a value just above 14% at 2.4 *f*. This trend clearly shows that there were fewer errors when measuring the resonance amplitude with higher camera speeds. In fact, the error was reduced by 50% when the camera speed rate was increased from 1.8 *f* to 2.4 *f*, indicating that faster sampling rates significantly improved the accuracy of the amplitude measurement.

#### 5.3.3. The Influence of the Camera’s Image Resolution on Resonance Amplitude

**Figure 21:**
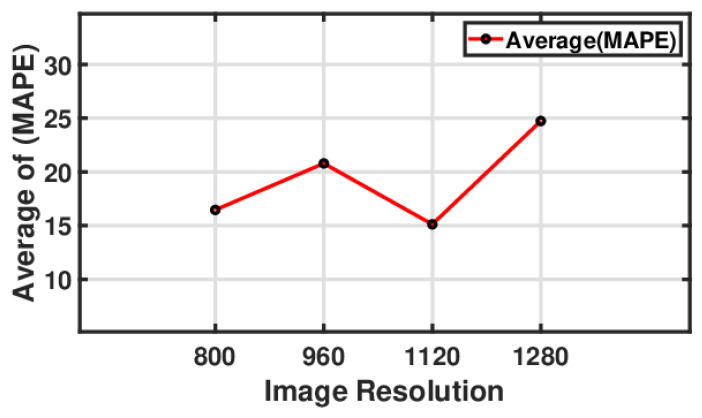
Amplitude of error prediction as a function of image resolution.

Figure 21 illustrates a line plot showing the relationship between the image-based vibration amplitude value and error percentage using different image resolutions. The average MAPE varied with changes in the image resolution. Initially, at an image resolution of 800, the average MAPE was close to 16. As the resolution increased to 960, the average MAPE increased to just over 20, reducing the prediction accuracy. However, when the resolution was increased to 1120, there was a significant reduction in the average MAPE, which dropped to just about 15, suggesting an improvement in the prediction accuracy. After this point, the average MAPE sharply increased to approximately 25 when the resolution reached 1280, indicating a significant decrease in the prediction accuracy at this higher resolution. This trend points out that there might be an optimal resolution of 1120 for the lowest prediction error, and deviations from this resolution—either lower or higher—lead to more significant prediction errors.

#### 5.3.4. Influence of Camera Image Resolution on Measured Natural Frequency

**Figure 22:**
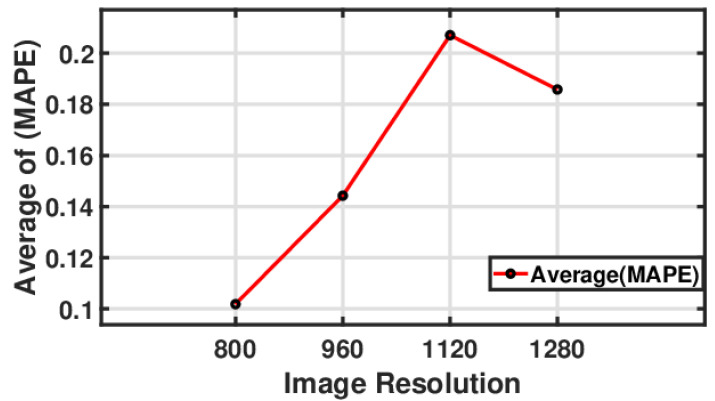
Average MAPE for natural frequency as a function of image resolution.

Figure 22 shows a line chart to show the relationship between the image-based frequency prediction value and the error percentage using different image resolutions. The image resolution had values of 800, 960, 1120, and 1280, while the average MAPE had values ranging from approximately 0.10 to 0.20. The observation is that as the image resolution increased from 800 to 1120, the average MAPE also increased, suggesting a positive correlation between the image resolution and the prediction error. However, upon increasing the resolution from 1120 to 1280, there was a sharp decrease in the average MAPE, indicating an improvement in the prediction accuracy. The lowest value of the average MAPE was at the image resolution of 800, which implies that the best prediction accuracy occurred at this resolution. The highest average of the MAPE was observed at a resolution of 1120, suggesting that increasing the resolution beyond 800 leads to a higher prediction error.

## 6. Modelling and Validation

The first step in developing a model includes the identity of the relation between the dependent variable and one or more independent variables. The regression equation was formulated using the MATLAB curve fitting feature to predict the dependent value based on the independent variables. Four empirical models were developed to predict the natural frequency based on four different image resolutions and to predict the resonance amplitudes. Moreover, an empirical model was developed to predict the crack depth on the beam structure.

This section describes how the model validation evaluated the performance and reliability of predictive models in this study. The experimental approach was designed to assess the quality of the model results of image-based dynamic response measurements of the beam structure and crack depth. First, the intact beam validation was prepared with three random thicknesses (3.7 mm, 5.3 mm, and 7.2 mm) for the purpose of predicting the image-based frequency and amplitude. Second, for the cracked beam, two random crack depth ratios (30%, 50%) were chosen 5 mm away from the fixed end of the beam. This deliberate choice of random thickness and crack depth ratios aimed to replicate the complexities observed in practical structural elements. The dynamic responses of the intact and cracked beams were measured and analysed through controlled experimental testing. These experimental data were further compared with the predictions generated by the empirical models, allowing for a rigorous validation process to evaluate the model’s accuracy in capturing the dynamic behaviour of beams with crack-induced structural variations.

### 6.1. Damping Ratio Modelling

Damping is one of the natural characteristics of all materials. With ABS, the molecular chains forming the fibres rub against one another, and the resulting friction causes energy loss, which has been observed as damping [35,81,82]. The impact tests were carried out three times to calculate the natural frequency and damping ratio obtained from the accelerometer and to calculate its averages. The two-input variables included in the first model equation were the beam thickness and damping ratio. The beam’s cross-sectional area increased as the thickness increased, leading to increased energy dissipation due to increased inter-filament surface friction. The test results indicate that as the thickness increased, the damping ratio increased. Different beam thicknesses from those initially examined were used to extend and develop the model.

**Figure 23:**
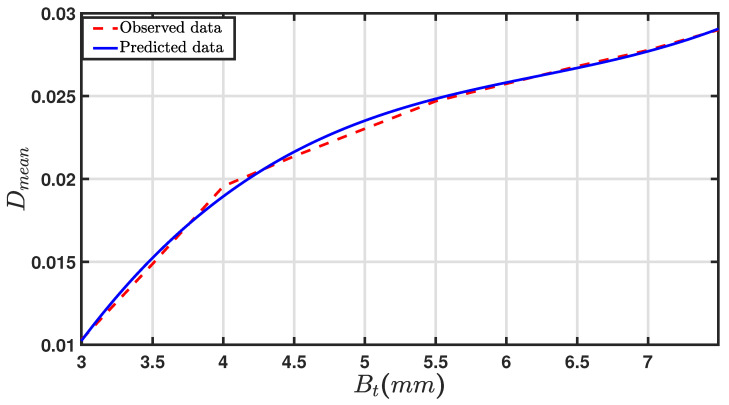
The relationship between beam thickness and damping ratio: measured and predicted.

Table 6 displays the mean damping ratio for different beam thicknesses. Figure 23 presents the predicted and observed data for the beam thicknesses and the mean damping ratio. The mean damping ratio $\left(D_{mean}\right)$ ranged from 0.0103 to 0.029. The mean damping underwent a more rapid change when the thickness was lower, transitioning from 0.0103 at 3 mm to 0.0196 for 4 mm. Thereafter, the damping ratio increased more gradually as the beam thicknesses increased.

Equation (4) shows the polynomial regression equation used to determine the correlation between the beam thickness and the mean damping ratio.

(4) $$D_{mean}=0.00031B_{t}^{3}-0.0058B_{t}^{2}+0.038B_{t}-0.0590,\;$$

where $D_{mean}$ is the damping ratio corresponding to the thickness $B_{t}$.

The thickness of the beam will impact its ability to dissipate energy due to the changes in its vibration-damping characteristics, i.e., changes in the independent variable, $\left(B_{t}\right)$, in the above, e.g., Equation (4) will change the value of $D_{mean}$.

The regression model is a cubic polynomial, reflecting how the damping ratio $D_{mean}$ is influenced by the beam thickness $\left(B_{t}\right)$. An R-squared value of 0.9980 indicates that the model is a good fit and reliable. The value of the root mean square error RMSE was $3.914\times 10^{-4}$.

### 6.2. Fundamental Frequency Modelling

The fundamental natural vibrational frequency of a beam can be calculated based on the material properties, geometry, and boundary conditions. The presence of damping affects the beam’s dynamic behaviour. Four different image resolutions of the camera were used to obtain the data to determine the fundamental frequencies of the given beam structures and provide data for modelling them. The models were used to investigate the accuracy of the image-based dynamic response measurements when predicting fundamental natural frequencies. The mean damping ratio $D_{mean}$ and image-based fundamental frequency $F_{cam}$ were used as independent variables for each image resolution to determine the value of $F_{acc}$, e.g., $F_{acc}=f\left(D_{mean},F_{cam}\right)$. Thus, for any of the given image resolutions, a measured value of $F_{cam}$ and a known value of $D_{mean}$ can be used to determine the value of $F_{acc}$ to a known level of accuracy.

**Figure 24:**
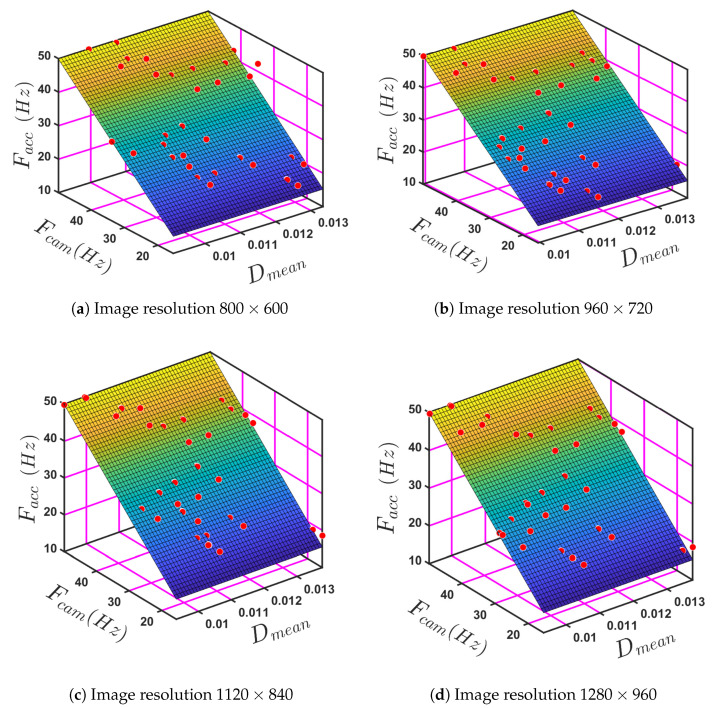
The 3D linear model to predict frequency $F_{acc}$ from $F_{cam}$ and $D_{mean}$ for four image resolutions.

Figure 24 displays a 3D surface plot for the Dmean, Fcam, and Facc for four different image resolutions: 800×600, 960×720, 1120×840, and 1280×960. The observed trends are shown in Figure 24a–d, where a linear relation can be seen for all four image resolutions. The curve generated to fit the data, Equation (5), applies to all four images.

(5) $$F_{acc}=P_{00}+P_{10}D_{mean}+P_{01}F_{cam},$$

where $F_{acc}$ is the frequency predicted to be measured by the accelerometer, $D_{mean}$ is the mean damping ratio, and $F_{cam}$ is the frequency measured by the camera and values of the coefficients $P_{00}$, $P_{10}$, and $P_{01}$ corresponding to each of the four resolutions are presented in Table 7.

**Figure 25:**
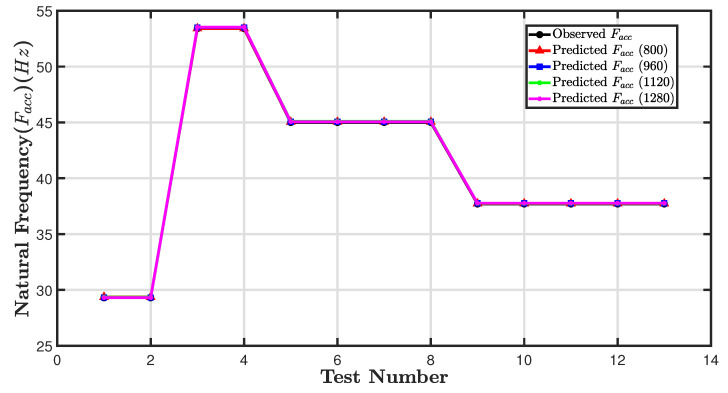
Comparison of validation and model prediction results for the $F_{acc}$.

Following the development of the empirical models, different beam thicknesses were employed to validate their reliability. The results of four beam thicknesses (3.7 mm, 4.5 mm, 5.3 mm, and 6.5 mm)—see Table 8—were compared to those used in the original empirical model. The results and the data obtained through experimentation are compared in Table 8 and Figure 25.

For each of the thirteen tests, the first was to measure the natural frequency of the four beams directly using the accelerometer, and the other thirteen were used to predict the natural frequency from $F_{cam}$ and $D_{mean}$. Based on Table 8, a close correspondence is observed between the experimental values for $F_{acc}$ and the model predictions obtained from $F_{cam}$ and $D_{mean}$ for all four values of image resolution. Figure 25 presents a visual comparison of the results of the model prediction and validation for $F_{acc}$. That is because fundamental frequency prediction has low sensitivity to image resolution. To evaluate the model’s accuracy, the RMSE was calculated for all four image resolutions (800×600, 960×720, 1120×840, and 1280×960) as 0.001693, 0.004047, 0.007868, and 0.005936, respectively, along with the mentioned R-squared values of 0.9883, 0.9883, 0.9888, and 0.9886.

### 6.3. Resonance Amplitude Modelling

The resonance amplitude of a beam can be defined as the maximum displacement of the beam under an applied force acting at the beam’s natural frequency. The material properties, geometry, and boundary conditions must be considered when measuring resonance amplitude. The resonance amplitudes obtained from the camera and accelerometer were used to develop a model for predicting resonance amplitude under a damped beam structure. As mentioned above, the same four different image resolutions were used when modelling the resonance amplitudes in beam structures. The resonance amplitude models were developed to explore the accuracy of image-based dynamic response measurements to predict resonance amplitudes in beam structures.

**Figure 26:**
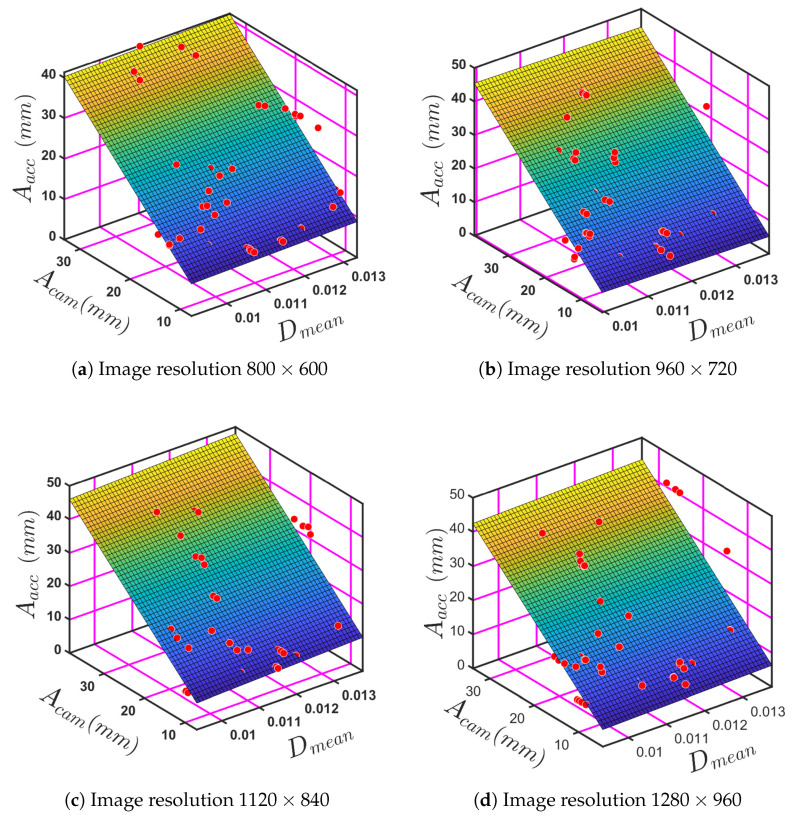
3D linear model to predict resonance amplitude $A_{acc}$ from $A_{cam}$ and $D_{mean}$ with four different image resolutions.

Figure 26 presents three-dimensional surface plots that illustrate the use of polynomial regression analysis. The relationship between the amplitude at resonance based on the accelerometer reading and mean damping ratio and the amplitude at resonance using each of the four image resolutions. The four resolutions show a linear correlation. The plots indicate that the amplitude, as measured by the accelerometer, decreases with an increase in the mean damping ratio, as would be expected. Moreover, the amplitude measured by the camera and the amplitude measured by the accelerometer appear to have a direct correlation. It can be concluded from the observed trends that there is a noticeable linear relationship, see Figure 26a–d across all image resolutions.

The Equation (6) generated applies to all four image resolutions and is represented as $A_{acc}$ = *f*($D_{mean}$, $A_{cam}$). Where the amplitude determined by the accelerometer is $A_{acc}$, and by the camera as $A_{cam}$, the mean damping ratio is $D_{mean}$. As above, the constant term is $P_{00}$, and the coefficients for $D_{mean}$ and $A_{cam}$ are $P_{10}$ and $P_{01}$ respectively.

(6) $$A_{acc}=P_{00}+P_{10}D_{mean}+P_{01}A_{cam}.$$

Table 9 displays the coefficients $P_{00}$, $P_{10}$ and $P_{01}$, with R-square values for different image resolutions.

The large variation of the coefficients with resolution, especially for $P_{10}$, indicates that image resolution impacts the amplitude prediction. The R-squared values indicate that the model fits the data well across the different resolutions. It is noted that as the image resolution increased, the R-square value decreased slightly.

**Figure 27:**
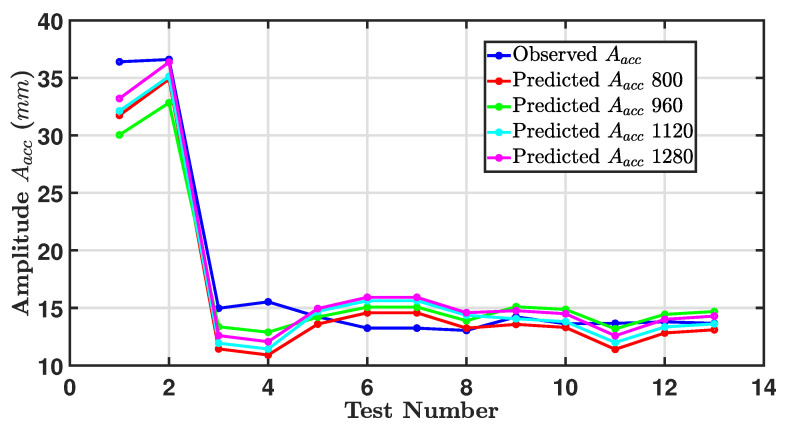
Comparison of validation and model prediction results for the $A_{acc}$.

Figure 27 shows a line graph comparing actual $A_{acc}$ and model prediction results for validation of the model. The graph has five lines representing observed $A_{acc}$ amplitude and four different predicted $A_{acc}$ amplitudes, one for each image resolution. It is clear that the predicted and actual values closely follow the same trend. A similar sharp decrease after the first test was followed by a general plateauing. The prediction model with higher resolutions (1120×840,960×720, 1120×840, and 1280×960) also closely follows the observed amplitudes with minor deviations.

In the tests from 2 to 13, all the predicted amplitudes follow the same trend, with slight variations in the exact values.

The prediction models at 1120×840 and 1280×960 resolutions (cyan and purple lines) are very close to each other across the tests. In contrast, models at 800×600 and 960×720 resolutions (red and green lines) show slightly more deviation from the observed values, especially towards the later tests.

To evaluate the model’s accuracy, RMSE was calculated for all four image resolutions, giving values of 5.3261, 5.8140, 4.8285 and 5.3463, respectively, and accompanied by an R-squared as tabulated in Table 10. Overall, the model predictions at different resolutions are relatively accurate but slightly underestimate the observed amplitude.

### 6.4. Modelling Crack Depth

The model also aims to accurately predict the depth of the crack in the ABS cantilever beam. A polynomial regression analysis model was used to show how independent variables crack depth $\left(C_{d}\right)$ and beam thickness $\left(B_{t}\right)$ affect the behaviour of the ABS beam using the damping ratio as the dependent variable. The relationship between these variables can be expressed as: $D_{mean}$ = *f*($C_{d}$,$B_{t}$). The experimental raw data indicate a nonlinear relationship between mean damping and beam thickness/crack depth. The nonlinear regression model form is shown in Equation (7):

(7) $$D_{mean}=P_{00}+P_{10}C_{d}+P_{01}B_{t}+P_{20}C_{d}^{2}+P_{11}C_{d}B_{t}+P_{02}B_{t}^{2}+P_{21}C_{d}^{2}B_{t}+P_{12}C_{d}B_{t}^{2}+P_{03}B_{t}^{3}.$$

**Figure 28:**
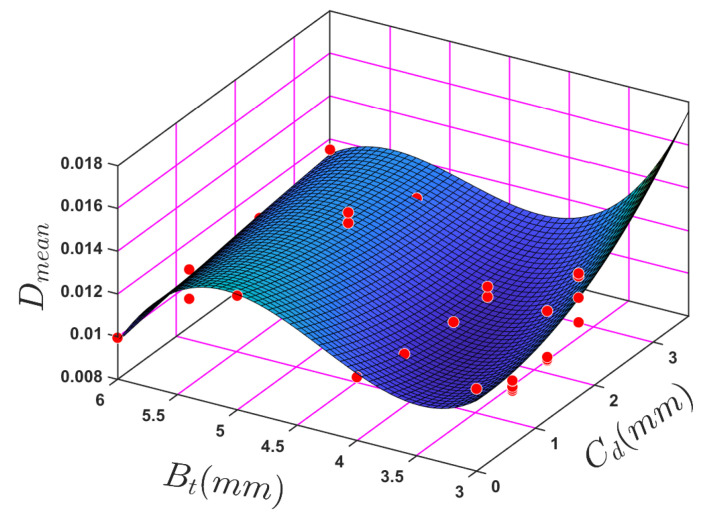
Experimental and surface fitted data for nonlinear empirical correlation between crack depth, beam thickness and mean damping ratio.

The variable $P_{00}$ to $P_{03}$ represent the regression coefficient. Here, in Figure 28, the *x*-axis was chosen to represent the crack depth and the *y*-axis to represent the beam thickness. In Figure 28, the fitted 3D surface has a relatively high R-squared value of 0.691.

Table 11 presents the fitted nonlinear regression coefficients. In order to predict crack depth, the polynomial formula in which the dependent variable $D_{mean}$ is replaced with Cd, giving a function of the form $C_{d}$ = *f*($B_{t}$,$D_{mean}$), where $B_{t}$ and $D_{mean}$ are the input independent variables. Symbolic algebra, which is extremely effective at solving complex equations, was used to solve for $C_{d}$. The command syntax to solve the equations for $C_{d}$ in MATLAB is as follows: Solve (‘The equation’, the variable that needs to be solved ‘$C_{d}$’). The user has to declare the other variables in the equations as symbolic in MATLAB.

Once the empirical model was established, various crack depths and beam thicknesses were used to validate its reliability with three beam thicknesses (3.5 mm, 4.5 mm, and 5.5 mm) were used with eight different crack depths. The independent variables were intentionally different from those used in the original empirical model. However, the regression analysis’s results of crack depth, beam thickness, and modal mean damping ratio yielded unsatisfactory results.

**Figure 29:**
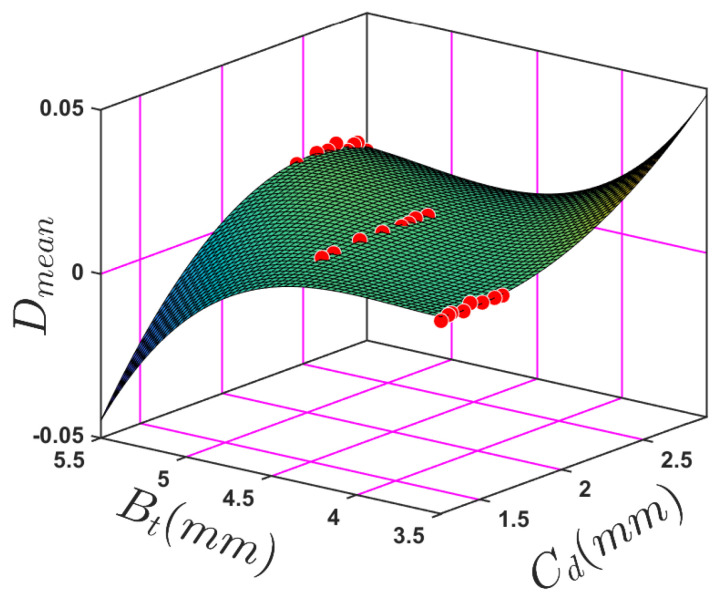
Nonlinear least squares are used to optimise empirical correlation between crack depth, beam thickness and mean damping ratio.

Figure 29 shows the nonlinear least squares (NLS) optimisation technique used to fit the model to the data by minimising the sum of the squares of the differences between the observed and predicted values. Once the optimisation was complete, the goodness of fit was evaluated using metrics such as the coefficient of determination (R-squared), RMSE, and NLS, depending on the specific context. Table 12 shows new fitting coefficients obtained using NLS.

**Figure 30:**
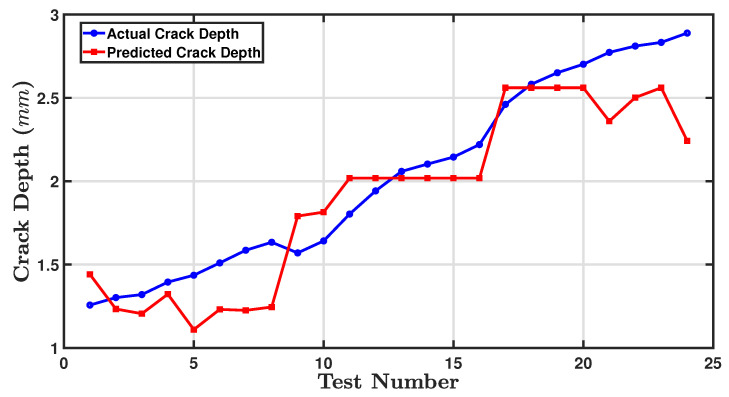
Comparison of model actual crack depth with predicted crack depth optimised with nonlinear least squares (NLS).

Performing validation by comparing the model’s predictions with experimental data is a crucial step after completing the empirical model to guarantee the accuracy and reliability of any model. Table 13 outlines validation data through a depiction of these particular parameters along with their resultant outcomes. Figure 30 compares the performance of measuring crack depth prediction after optimisation against the actual recorded measurements.

Figure 30 compares the performance of measuring crack depth prediction after optimisation against the actual recorded measurements. The actual crack depth values (blue line) increase gradually over the test numbers because of the ratio of the crack depth based on the beam thickness, indicating a trend of growing crack depth as the test number increases. The red line NLS generally follows the same trend but with less fluctuation, suggesting that this method smooths out some of the variability seen in the actual measurements. Overall, the nonlinear least squares method seems to track more closely with the actual crack depth measurements than the predicted crack depth method, showing some variance from the actual data measurements.

The actual crack depth (blue line) gradually increases with the test number because the ratio of the crack depth to beam thickness increases as the test number increases. The red line obtained using NLS generally follows the same trend but with less fluctuation, suggesting that this method smooths out some of the variability seen in the actual measurements. Overall, the NLS method seems to track the actual crack depth measurements than the predicted crack depth method, showing some variance from the actual data measurements.

## 7. Error Discussion

After developing the prediction frequency, amplitude, and crack depth models, the following stage was to validate their accuracy and predictive capabilities. The first step was to assess the model predictions against the experimental points.

**Figure 31:**
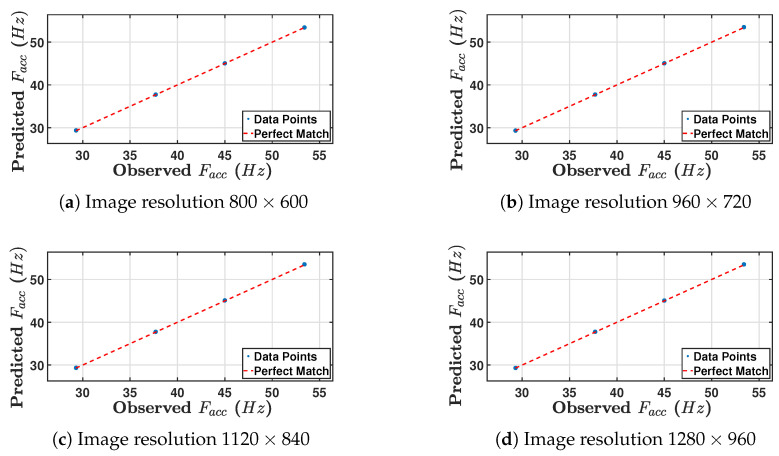
Observed and predicted ($F_{acc}$) values for four different image resolutions.

The first step was to assess the model predictions against the experimental points. Figure 31 shows four scatter plots for four different image resolutions of observed and predicted ($F_{acc}$) values. The blue dots represent observations against predicted measurements. The dashed red line represents a perfect match, i.e., of the form (y=x). Table 14 presents the mean absolute percentage error for each image resolution for the $F_{acc}$ model.

**Figure 32:**
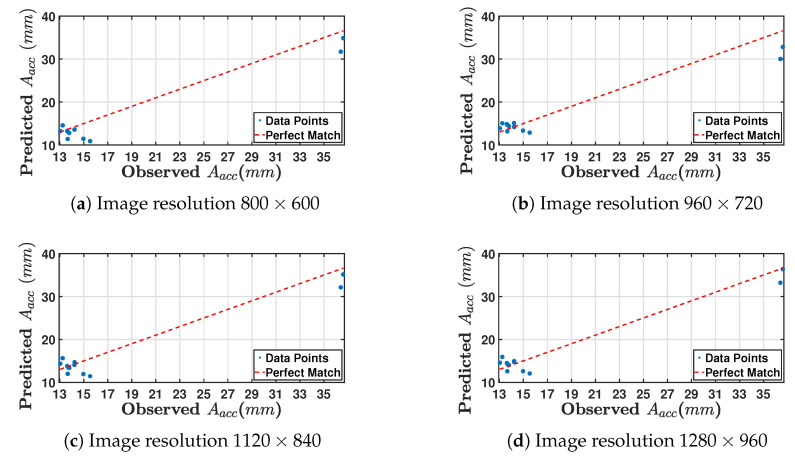
Four different image resolutions of observed ($A_{acc}$) and predicted ($A_{acc}$) values.

All the actual data points and predicted values cluster closely around the diagonal, which indicates that four models of $A_{acc}$ using different image resolutions performed very well, making accurate predictions. Figure 32 presents four scatter plots for comparable observed and predicted values of $A_{acc}$ in mm for four different image resolutions. Table 15 provides the mean absolute percentage error for each image resolution for the $F_{acc}$ model; when compared with Table 14. The amplitude prediction errors are significantly greater. This indicates that imaging device settings and boundary conditions influence amplitude measurement.

Figure 32a shows data points for the 800 × 600 resolution clustered near the lower left of the graph, indicating that both observed and predicted values of $A_{acc}$ are relatively low. The points deviate from the perfect match line, suggesting that the predictions are not highly accurate at this resolution. There are no observed or predicted values exceeding 27 mm.

Figure 32b presents data points of 960 × 720 resolution where the predicted values are consistently higher than the observed values, indicating a tendency to overestimate $A_{acc}$. Some predicted points show significant errors, far from the perfect match line.

Figure 32c shows data points of the $A_{acc}$ model for 1120×840 resolution. As with the 800 × 600 resolution plot, the data points are primarily low values clustered near the lower left, though with more spread compared to the 800 × 600 resolution. However, while the predictions still deviate significantly from the perfect match line, this resolution indicates a slightly better prediction accuracy than the 800× 600 resolution.

Figure 32d shows that the data points for model $A_{acc}$ of 1280×960 resolution are spread out, but there is a noticeable improvement in prediction accuracy. The points are closer to the perfect match line than the other three resolutions, though there are still a few points that deviate significantly.

The choice of exposure time, as 1000 μs, could influence the measurements of the amplitude of vibrating beams. However, using shorter exposure times is less than the period of the motion and thus may not capture the full range of vibration amplitude. The room light was not bright enough, and the camera automatically set the ISO. This can lead to a degraded image quality, which can cause your photos to be grainy or noisy. It will, therefore, also decrease the internal noise of the image sensor. Accurate measurements of vibration amplitude are a complex task that requires balancing the need to ‘freeze’ the motion and capture the full range of vibration amplitudes. The challenge is further complicated because higher frequencies can produce more noise in the images, impacting the image-based amplitude measurements.

**Figure 33:**
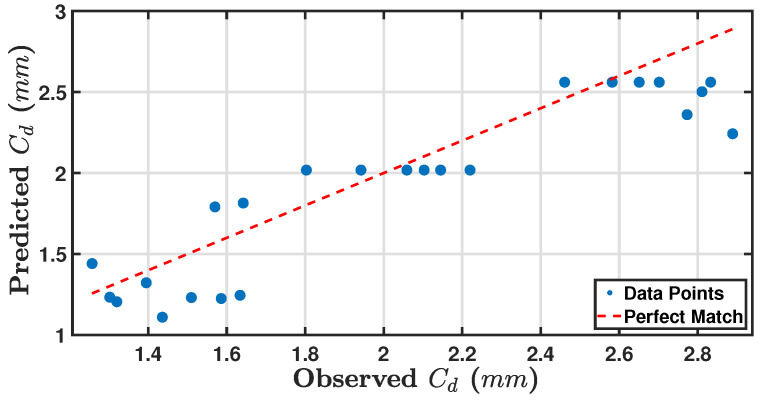
The observed $C_{d}$ and predicted $C_{d}$ measurements.

Figure 33 shows a comparison between observed and predicted $C_{d}$ values. The data points show a good alignment with the perfect match line, but noticeable variations exist, as with 2.889 mm observed and 2.246 mm predicted. The spread of data points indicates that the model has a reasonable but not perfect predictive capability across the observed range.

## 8. Conclusions

This study seeks to fill the gap to explore the potential of image-based dynamic response measurement to quantitatively assess the small or hidden cracks of small sizes in beam structures. The research hypothesis is that different image resolutions and camera speed rates will affect the accuracy of dynamic response measurements of fundamental frequency and resonance amplitude. The dynamic response results obtained from the accelerometer were compared with those derived from image-based determinations of dynamic responses. These models aim to evaluate which camera settings yield better results in predicting the dynamic responses of the ABS beam in the presence of a small crack.

The test rig for the research experimental setup was built and checked. The specimens to be tested were four beam thicknesses with four crack depths and combinations of four camera settings (frame rate and resolution). The experimental setup was successfully used to conduct the test scenarios designed to explore the potential impact of imaging device parameters on the accuracy of dynamic response measurements in different damped structures. The results of experiment data were verified through comparison with empirical models, providing a reliable basis for our findings.

Experimental setups were developed based on the proposed experimental research scenarios. The nonlinear regression model was used to investigate the effects of damping on the beam structure, considering crack depth and beam thickness. The model’s initial results needed to be improved. The model was optimised using nonlinear least squares optimisation. The symbolic algebraic method was used to solve the nonlinear regression model’s formula to predict crack depth. The final results of the prediction were appropriate and satisfactory.

The developed models were validated using beam thicknesses and crack depths that were not considered during model development. Error analysis was carried out, and the accuracy of predicted versus measured crack depths was discussed.

The study has investigated how changes in camera resolution impact the accuracy of image-based measurements of a beam’s dynamic response. Based on four image resolutions, models have been created to examine the influence of resolution on the accuracy of the dynamic response, to assess which camera resolutions are best suited for accurately capturing the structure’s dynamic response. The dynamic responses empirical models result of four image resolutions were verified through comparison with the results of experimental data. The fitting accuracy for predicting of natural frequency is over 98% for all resolutions. The fitting accuracy for predicting resonance amplitude is around 90% for all resolutions excluding 1280×960 is less. The frame rate speed was assessed by comparing dynamic response measurements from the camera with those from the accelerometer.

The correlation between camera frame rate and dynamic response has been investigated. One research goal was to understand how changes in frame rate impact the camera’s accuracy in capturing the beam’s dynamic response. Camera speed rates were set based on a multiplier of the nature frequency of beam structure of these values 1.8, 2, 2.2 and 2.4. Models were created to examine the influence of frame rate on the accuracy of the captured dynamic response. The aim was to assess which camera frame rates are best suited for accurately capturing the structure’s dynamic response. The best frame per second for measuring natural frequency and amplitude was 2.4 of the beam’s natural frequency. The findings provide a damage assessment method by establishing an empirical relationship that predicts the crack depth based on the beam thickness and damping.

Modelling and Quantifying the Influence of Structural Damping on Crack Depth and Beam Thickness. The model was developed through a series of experiments and data collection involving structural damping, crack depth, and beam thickness. Nonlinear regression was used to analyse the correlations between these variables, which were identified and translated into equations to determine their relationships. The model was optimised using least squares optimisation. The model’s reliability provides valuable insights into the influence of structural damping on crack depth and beam thickness. The findings provide a damage assessment method by establishing an empirical relationship that predicts the crack depth based on the beam thickness and damping.

Based on the findings, there are several suggestions for future research. It is important to carefully choose and optimize the camera setup and efficiently manage data to enhance accuracy. Examining non-resonant conditions and varying frequencies can provide a more comprehensive understanding of structural behaviour in different dynamic scenarios. In addition, future studies should consider employing a variety of materials. The principles and methods used can be expanded to other polymeric materials with similar mechanical properties, such as PLA (Polylactic Acid) and PETG (Polyethylene Terephthalate Glycol-modified). Exploring various mechanical properties like steel and aluminium, as well as different beam geometries, can improve the applicability of the results. It is also crucial to validate the developed model across a wider range of crack depths and positions not previously considered, as this could significantly enhance its accuracy and reliability. Comparing the camera-based measurements with those from other non-contact methods, such as laser Doppler vibrometry, would be beneficial in reinforcing the findings.

This study acknowledges several limitations that could impact the generalizability and accuracy of the findings. One key limitation is the influence of environmental factors, such as temperature, humidity, lighting conditions, and shadows, on camera-based measurements. These factors can introduce variability and affect the precision of results in practical applications. Additionally, the distance between the camera and the object being measured was not extensively explored, potentially leading to uncertainties when applying the findings to different setups or conditions. Addressing these limitations in future research will be crucial for enhancing the robustness and applicability of camera-based measurement techniques.

## Figures and Tables

**Table 1:** Ultimaker Cura 4.0 ABS filament specifications.

Filament Specification	Value
Diameter	2.85 ± 0.10 mm
Tensile modulus	1618.5 MPa (ISO 527) [50]
Vicat softening temperature	97 °C
Elongation at yield	3.5% (ISO 527)
Vicat softening temperature	225–245 °C
Flexural modulus	2070 MPa (ISO 178) [50]

**Table 2:** Printing parameters.

Parameters	Value
Nozzle diameter	0.4 mm
Printing Orientations	0°
Layer Thickness	0.5 mm
Infill density	100%

**Table 3:** The experimental scheme of the dynamic response tests.

Beam Thickness from 3 mm to 6 mm with 1 Steps Increment		
Crack Depth Ratio	FPS = *n* × Fundamental Frequency (*f*)	Image Resolution
0%	1.8×f	800×600
20%	2×f	960×720
40%	2.2×f	1120×840
60%	2.4×f	1280×960

**Table 4:** Mean damping ratio $D_{mean}$ for different crack ratios in an ABS cantilever beam.

Beam Thickness (mm)	Mean Damping Ratio $D_{\mathrm{mean}}$			
	0% $C_{d}$	20% $C_{d}$	40% $C_{d}$	60% $C_{d}$
3	0.0119	0.0108	0.0114	0.0130
4	0.0110	0.0104	0.0100	0.0102
5	0.0133	0.0121	0.0133	0.0111
6	0.0099	0.0101	0.0107	0.0115

**Table 5:** Mean fundamental frequency $F_{\mathrm{mean}}$ for different crack depth ratios and thicknesses of ABS cantilever beam.

Beam Thickness (mm)	Mean Fundamental Frequency $F_{\mathrm{mean}}$			
	0% $C_{d}$	20% $C_{d}$	40% $C_{d}$	60% $C_{d}$
3	24.60	23.33	21.83	18.71
4	32.67	32.01	30.59	28.33
5	42.26	41.52	40.64	33.90
6	49.90	49.68	46.39	41.52

**Table 6:** Beam thickness and mean damping ratio.

Beam Thickness ($B_{t}$) (mm)	Mean Damping Ratio ($D_{\mathrm{mean}}$)
3.0	0.0103
3.5	0.0149
4.0	0.0196
4.5	0.0214
5.5	0.0247
6.5	0.0268
7.0	0.0278
7.5	0.0290

**Table 7:** Coefficients and R-square values for different image resolutions.

Image Resolution	$P_{00}$	$P_{10}$	$P_{01}$	R-Square
800×600	0.002492	7.836	0.9989	0.9883
960×720	0.04552	−6.494	1.002	0.9883
1120×840	−0.1418	5.982	1.004	0.9888
1280×960	0.05718	−10.59	1.003	0.9886

**Table 8:** Observed and predicted $F_{acc}$ values.

Test Number	Beam Thickness	Observed $F_{\mathrm{acc}}$	Predicted $F_{\mathrm{acc}}$ 800	Predicted $F_{\mathrm{acc}}$ 960	Predicted $F_{\mathrm{acc}}$ 1120	Predicted $F_{\mathrm{acc}}$ 1280
1	3.7	29.3	29.365659	29.325061	29.348226	29.316156
2	3.7	29.3	29.365659	29.325061	29.348226	29.316156
3	6.5	53.4	53.413058	53.494883	53.524708	53.523716
4	6.5	53.4	53.413058	53.494883	53.524708	53.523716
5	5.3	45.4	45.043402	45.060593	45.107219	45.069995
6	5.3	45.0	45.043402	45.060593	45.107219	45.069995
7	5.3	45.0	45.043402	45.060593	45.107219	45.069995
8	5.3	45.0	45.043402	45.060593	45.107219	45.069995
9	4.5	37.7	37.73321	37.761095	37.764108	37.772721
10	4.5	37.7	37.73321	37.761095	37.764108	37.772721
11	4.5	37.7	37.73321	37.761095	37.764108	37.772721
12	4.5	37.7	37.73321	37.761095	37.764108	37.772721
13	4.5	37.7	37.73321	37.761095	37.764108	37.772721

**Table 9:** The coefficients $P_{00}$, $P_{10}$ and $P_{01}$, with R-square values for different image resolutions.

Image Resolution	$P_{00}$	$P_{10}$	$P_{01}$	R-Square
800×600	−3.66	202.3	1.269	0.8765
960×720	3.479	−217.0	1.125	0.8765
1120×840	−4.751	455.3	1.207	0.8752
1280×960	−3.157	270.3	1.274	0.8136

**Table 10:** Observed and predicted $A_{acc}$ values.

Test Number	Beam Thickness	Observed $A_{\mathrm{acc}}$	Predicted $A_{\mathrm{acc}}$ 800	Predicted $A_{\mathrm{acc}}$ 960	Predicted $A_{\mathrm{acc}}$ 1120	Predicted $A_{\mathrm{acc}}$ 1280
1	3.7	36.3988337	31.746073	30.042207	32.125615	33.206715
2	3.7	36.59950842	34.893194	32.832206	35.118978	36.366237
3	6.5	14.97032404	11.44107	13.360975	11.93737	12.597951
4	6.5	15.5288699	10.90809	12.888475	11.43043	12.062871
5	5.3	14.23290136	13.597539	14.205296	14.696484	14.94391
6	5.3	13.2449246	14.574669	15.071546	15.625874	15.92489
7	5.3	13.2449246	14.574669	15.071546	15.625874	15.92489
8	5.3	13.04211282	13.242219	13.890296	14.358524	14.58719
9	4.5	14.22441142	13.571248	15.10367	14.060152	14.761238
10	4.5	13.62252477	13.304758	14.86742	13.806682	14.493698
11	4.5	13.66225308	11.401258	13.17992	11.996182	12.582698
12	4.5	13.7921466	12.822538	14.43992	13.348022	14.009578
13	4.5	13.66146181	13.101718	14.68742	13.613562	14.289858

**Table 11:** The regression coefficients for nonlinear regression correlation between crack depth, beam thickness and mean damping ratio.

Coefficients	Value
$P_{00}$	0.1098
$P_{10}$	0.01085
$P_{01}$	−0.07406
$P_{20}$	0.001111
$P_{11}$	−0.005649
$P_{02}$	0.01791
$P_{21}$	−0.0001901
$P_{12}$	0.0006547
$P_{03}$	−0.001392

**Table 12:** New fitting Coefficients obtained by using Nonlinear least squares.

Coefficients	Value
$P_{00}$	0.2763
$P_{01}$	−0.2383
$P_{02}$	0.0881
$P_{03}$	−0.01106
$P_{10}$	0.1084
$P_{11}$	−0.1472
$P_{12}$	0.02888
$P_{20}$	0.1095
$P_{21}$	−0.02603

**Table 13:** Experimental and predicted crack depth with nonlinear least squares.

Beam Thickness (mm)	Actual Crack Depth (mm)	Crack Depth Using *NLS* (mm)
3.5	1.257	1.441152
3.5	1.302	1.648801
3.5	1.32	1.676815
3.5	1.395	1.55972
3.5	1.436	1.772248
3.5	1.51	1.65104
3.5	1.586	1.656967
3.5	1.634	1.636961
4.5	1.57	2.245666
4.5	1.642	2.2219
4.5	1.803	2.018337
4.5	1.942	2.018337
4.5	2.059	2.018337
4.5	2.103	2.018337
4.5	2.145	2.018337
4.5	2.22	2.018337
5.5	2.461	2.56082
5.5	2.582	2.56082
5.5	2.651	2.56082
5.5	2.702	2.56082
5.5	2.773	2.761254
5.5	2.811	2.619452
5.5	2.833	2.56082
5.5	2.889	2.87912

**Table 14:** Mean absolute percentage error for image resolution for $F_{acc}$ model.

Image Resolution	MAPE (%)
800×600	0.1018
960×720	0.1443
1120×840	0.2071
1280×960	0.1858

**Table 15:** Mean absolute percentage error for image resolution of $A_{acc}$ models .

Image Resolution	MAPE (%)
800×600	10.112
960×720	9.2959
1120×840	10.0010
1280×960	9.9332

## Data Availability

Data are contained within the article.
